# Research progress on the mechanism underlying the application of mesenchymal stem cells in the treatment of male infertility

**DOI:** 10.3389/fendo.2025.1671247

**Published:** 2025-09-11

**Authors:** Xingzhao Tian, Jingyi Zhang, Xinyi Tang, Xinlei Guo, Yanan Gong, Fang Yang, Liang Dong, Xujun Yu

**Affiliations:** ^1^ Traditional Chinese Medicine (TCM) Regulating Metabolic Diseases Key Laboratory of Sichuan Province, Hospital of Chengdu University of Traditional Chinese Medicine, Chengdu, China; ^2^ School of Medical and Life Sciences, Chengdu University of Traditional Chinese Medicine, Chengdu, China

**Keywords:** mesenchymal stem cells, male infertility, testicles, sperm, regenerative medicine, mesenchymal stem cells-derived exosomes

## Abstract

Male infertility has become an increasingly prominent health issue worldwide. This review systematically evaluated the therapeutic application of different types of MSCs in various male infertility models. The therapeutic effects of MSCs are attributed to mechanisms such as *in vivo* and *in vitro* differentiation into germ cells, improved antioxidant capacity of testicular tissue, inhibited secretion of inflammatory factors and elevated anti-inflammatory level of testicular tissue, prevention of excessive apoptosis of testicular tissue cells, restoration of the normal secretion of sexual hormone levels *in vivo*, and regulation of sperm autophagy. Simultaneously, this study also emphasized on the latest progress in the research of MSC-Exos, with the discussion of its potential advantages over traditional MSC therapy. In addition, this review also elucidated challenges in the clinical translation of MSCs, including safety and standardization issues, as well as the necessity of conducting human clinical trials. On these basis, this research proposed corresponding improvement plans, such as developing engineered MSCs products, optimizing delivery methods and exploring combination therapies, which may provide potential reference for the clinical application of MSCs and MSC-Exos on a large scale.

## Graphical Abstract

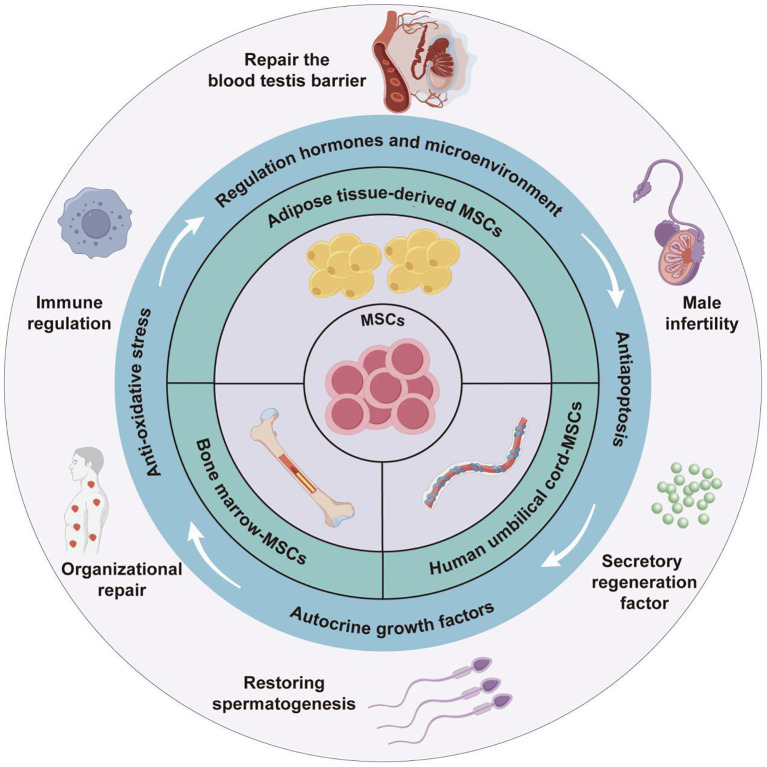

## 1 Introduction

Male infertility has become an increasingly serious health issue worldwide recently, which may involve around 2.5% -12% of the global male population (1). The affected males may usually present with reduced sperm count, decreased sperm motility, or loss of fertilization ability (2). At present, the treatment of male fertility has been benefited a lot by the assisted reproductive technology and drug therapy (3). However, existing therapies are still insufficient for infertility caused by testicular and other reproductive organ lesions or failures (4). Stem cell (SC) therapy has emerged has a novel therapeutic solution with great potential in treating male infertility. Therefore, it underscores the great importance of applying SC therapy to reshape the male reproductive microenvironment and effectively intervene in sperm production for the treatment of refractory male infertility.

SCs are widely present in embryos, fetuses, and adult tissues, which are a type of undifferentiated primitive cells with multipotent differentiation potential. SCs are mainly divided into three categories of embryonic SCs, induced pluripotent SCs, and adult SCs based on their sources and characteristics (5). Acting as one of the most promising applications in regenerative medicine, SC therapy has been widely applied and studied for the treatment of various diseases, such as musculoskeletal and neurological disorders, immune disorders, blood dysfunction, and degenerative diseases (6). Among them, mesenchymal SCs (MSCs) stand out in the past two decades due to their widespread distribution, strong tissue repair ability, and significant immune regulatory properties (7). For example, in terms of their immune regulation, MSCs could upregulate the expression of CD24, converting activated neutrophils into senescent neutrophils, thereby reducing chemotaxis, reactive oxygen species (ROS) production, nicotinamide adenine dinucleotide phosphate (reduced form) oxidation, and granule secretion, ultimately mitigating acute lung injury (8). As for tissue repair, MSCs could effectively increase the length of the epithelial margin, collagen content and micro-vessel density of the wound bed of rats, and promote the expression of vascular endothelial growth factor (VEGF), thereby accelerating the healing of ischemic wounds in rats with diabetes through systemic or local administration (9). Meanwhile, MSCs outperform other types of SCs for their good isolation characteristics, lower toxicity and side effects, and less ethical controversy (10). Furthermore, with the deepening of research on MSCs in recent decades, MSC-derived exosomes (MSC Exos), are highly concerned as an important mediators of intercellular communication, and have been documented to possess therapeutic properties similar to parental cells, and, to some extent, avoid adverse reactions such as infusion toxicity (11). In the field of research on male infertility, great attention has been attached to the potential roles of MSCs and their extracellular vesicles (EVs) gradually. For example, bone marrow-derived MSCs (BM-MSCs) can increase the proportion of G2-M phase mitotic cells in testicular cells, maintain cell viability, promote the expansion of seminiferous tubules and cavities, thereby promoting the survival, expansion, and differentiation of spermatogonial SCs. Moreover, BM-MSCs can also promote the *in vitro* maturation of testes before puberty, providing a potential therapeutic option for chemotherapy-induced childhood infertility (12). In addition, under the induction of retinoic acid (RA) and testosterone (T), adipose-derived MSCs (ADSCs), through co-culturing with Sertoli cells, can activate transforming growth factor β (TGFβ)-SMAD family member 2/3, Janus kinase 2-signal transducer and activator of transcription 3 and phosphoinositide 3-kinase-protein kinase B (AKT) signaling pathway, thereby effectively stimulating the differentiation of ADSCs into male germ cells *in vitro* (13). Therefore, MSCs and their EVs exhibit unique advantages and broad application prospects in the treatment of male infertility, underlining the great significance of summarizing the related research progress comprehensively. Accordingly, this study was designed to review the latest research progress of MSCs and their exosomes (Exos) in treating male infertility, and to deeply explore the functional mechanisms of MSCs from different sources and their enormous potential in improving spermatogenesis, enhancing sperm quality, repairing testicular tissue damage, and *in vitro* and *in vivo* differentiating into germ cell. This study also discussed about the key roles of MSC Exos in intercellular signaling and their unique advantages in avoiding cell therapy-related side effects such as immune rejection and infusion toxicity. Through such comprehensive analysis of current research results, this study is anticipated to provide theoretical basis for the development of appropriate therapeutic strategies for male infertility, to explore the translational potential of MSCs and their EVs in regenerative medicine, and to offer new research ideas for future basic research and clinical applications.

## 2 Classification and characteristics of MSCs

**Figure 1:**
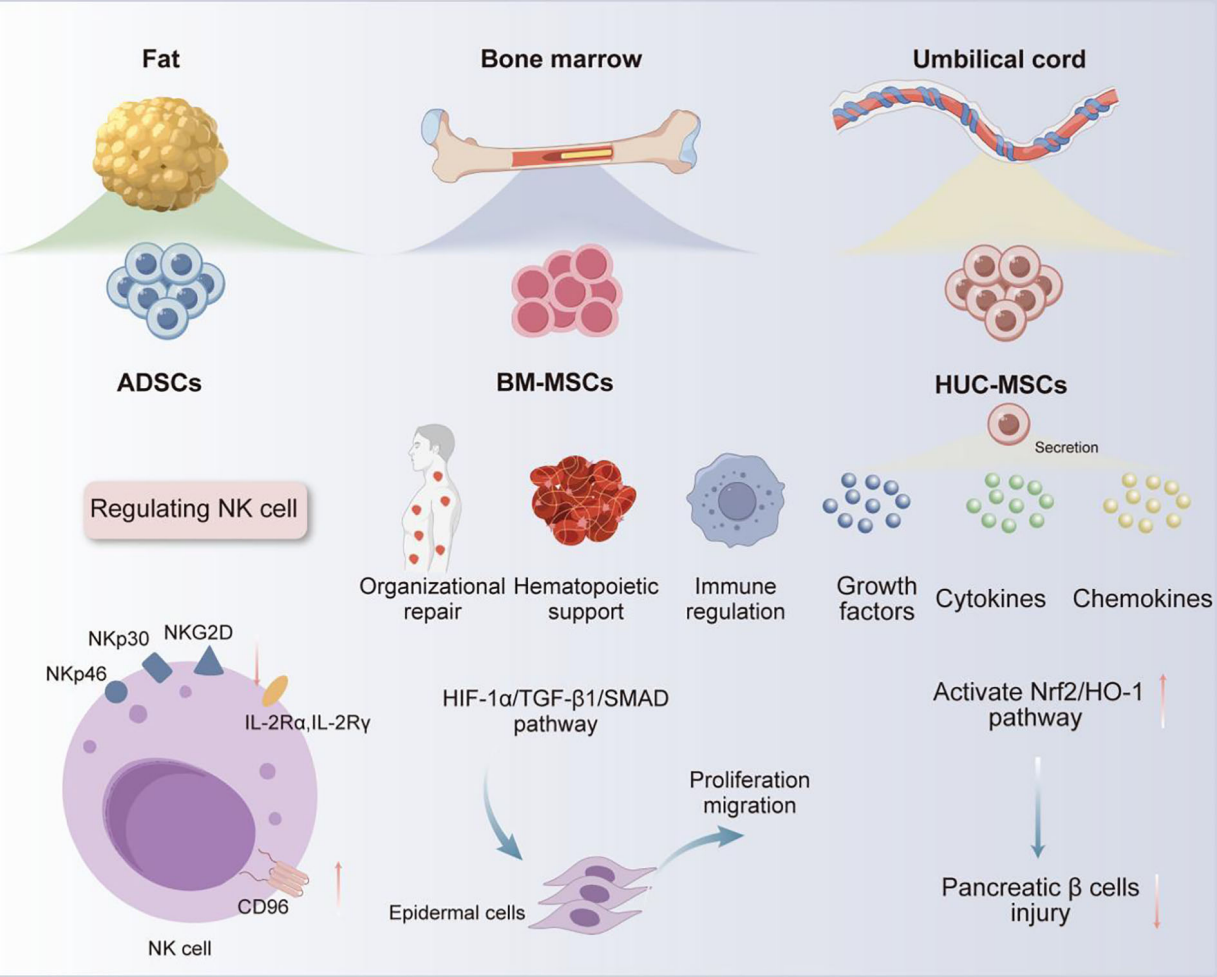
Classification and characteristics of commonly applied MSCs.

MSCs are a type of multipotent progenitor cells enabling self-renewal *in vitro* and differentiation into various MSCs, which are critical for exerting their therapeutic roles in tissue regeneration, immune regulation, anti-inflammatory, and wound healing (10). MSCs are widely present in various tissues of the human body, including peripheral blood, dental pulp, bone marrow, fat, umbilical cord, amniotic fluid, and placenta (6, 14). Among them, bone marrow, adipose tissue, and umbilical cord tissue are currently the main sources of MSCs for clinical application (15). BM-MSCs are renowned for their outstanding multi-directional differentiation ability, which are essential in facilitating tissue repair (16), immune regulation (17), hematopoietic support (18), etc. For example, in tissue repair, under low oxygen conditions, BM-MSCs could promote autophagy of epidermal cells, and enhance the proliferation and migration of epidermal cells by activating hypoxia-inducible factor-1α/TGFβ 1/SMAD family member signaling pathway, providing a novel strategy for diabetic wound treatment (19). However, the clinical application of BM-MSCs is still restricted by limitations such as highly invasive acquisition methods, unsustainable differentiation cycles, and tumorigenic risks (20). In contrast, ADSCs have significant advantages in aspects of accessibility, cellular activity, and proliferation ability, with demonstrated better safety and efficacy in the treatment of autoimmune diseases (21). ADSCs have been reported to enable the upregulation of Cluster of Differentiation (CD) 96, which is the inhibitory receptor of natural killer (NK) cells, while downregulating the expression of their activated receptors (e.g., NK group 2D, NK p30, and NK p46) and receptor subunits of interleukin-2 (IL - 2) (IL - 2 receptor α, IL - 2Rα, and IL - 2 receptor γ, IL - 2Rγ). By upregulating IL - 2 mediated negative regulatory factors (e.g., cytokine inducible SH2-containing protein) and dual specificity protein phosphatase 4 signaling pathway, ADSCs can effectively inhibit IL - 2-mediated NK cell effector function, but without impact on NK cell proliferation. Based on this mechanism, ADSCs can be useful for treating autoimmune diseases caused by excessive activation of NK cells (22). Moreover, human umbilical cord MSCs (HUC-MSCs) have recently become a research hotspot for their abundant sources, low ethical controversies and infection risk, as well as strong proliferation and differentiation abilities (23, 24). HUC-MSCs have been demonstrated to secrete various growth factors, cytokines, and chemokines (25), exhibiting significant therapeutic effects in lung diseases (26), skin diseases (27), neurological diseases (28), digestive system diseases (29), etc. Besides, the potential of HUC-MSCs in the treatment of diabetes and its complications has gradually emerged with the deepening of research (30–32). For example, Liu et al. found that intravenous injection of HUC-MSCs could effectively alleviate high glucose-induced oxidative stress, and protect pancreatic beta cells from damage by activating nuclear factor erythroid 2-related factor 2/heme oxygenase-1 signaling pathway. Therefore, HUC-MSCs can be used to treat type I diabetes caused by pancreatic beta cell damage (**Figure 1**) (27). With respect to the above, MSCs from different sources have exhibited unique functional mechanisms and significant therapeutic effects of in the treatment of various diseases, especially their advantages in cell regeneration, immune regulation, and tissue repair. All these characteristics support the vast potential of MSCs in male infertility treatment (**Table 1**).

**Table 1:** Classification and characteristics of commonly applied MSCs.

	ADSCs	BM-MSCs	HUC-MSCs
Surface markers	CD13, CD90, CD105, and STRO - 1 (33)	CD90, CD105, CD146, and CD271 (34)	CD44, CD73, CD90, and CD105 (35)
Treatment characteristics	Tissue regeneration (36), immune regulation (37), tissue repair (38), and anti-aging (39)	Organizational repair (16), immune regulation (17), and hematopoietic support (18)	Immune regulation (40), and tissue repair (41)
Clinical application	Skeletal diseases (42), cardiovascular diseases (38), kidney diseases (43), liver diseases (44), ophthalmic diseases (45), and skin diseases (46)	Skeletal diseases (47), cardiovascular diseases (48), lung diseases (49), kidney diseases (50), and neurological diseases (51)	Liver disease (52), lung disease (53), neurological disease (54), kidney disease (55), and bone disease (56)
Advantage	Rich sources, low ethical controversy, low risk of infection, high proliferation and differentiation ability (23, 24)	Mature research, sufficient sources, low immunogenicity, and few ethical issues (57, 58)	Convenient access, strong proliferation ability, low immunogenicity, and minimal ethical controversy (59)

## 3 Application of MSCs from different sources in the treatment of male infertility

### 3.1 Adipose-derived stem cells

**Figure 2:**
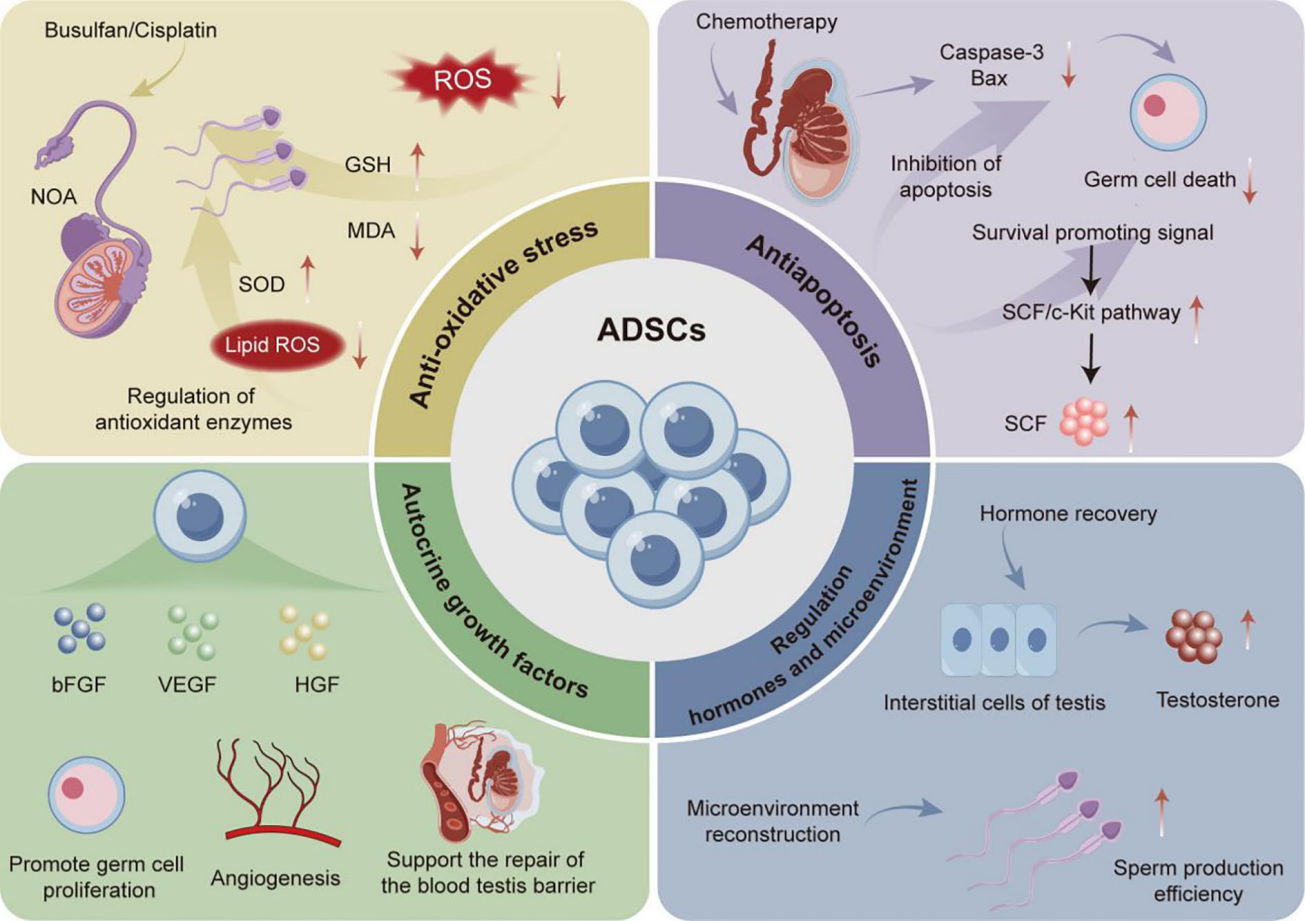
Application of ADSCs in the treatment for male infertility.

The application of ADSCs in the treatment of various diseases has attracted widespread research attention worldwide (60). There have been a series of experimental studies on the application of ADSCs to treat various types of male infertility. Among them, ADSCs exhibit therapeutic characteristics that can effectively restore male reproductive ability through multiple mechanisms for male infertility induced bythe use of chemotherapic agents, varicocele, and testicular torsion (**Figure 2**).

It has been reported that chemotherapic agents commonly administrated in cancer treatment could cause toxic damage to the reproductive system, which is inevitable in this process and is also an important factor leading to nonobstructive azoospermia (NOA) in the clinical setting (61) For example, Busulfan and cisplatin, two common chemotherapic agents in cancer treatment, may trigger significant side effects including damage to testicular tissue structure and function, leading to decreased fertility and, in severe cases, inducing NOA (62, 63). It highlights an urgent need for novel therapeutic schemes to overcome the toxicity of chemotherapic agents to the reproductive organs and reshape the damaged reproductive microenvironment. Existing studies have documented that a single-dose injection of ADSCs at a specific quantity can significantly enhance the recovery of chemotherapy-induced reproductive impairment across various experimental animal models through multiple mechanisms. For instance, in related experiments, Ganjibakhsh et al. induced azoospermia in mice via intraperitoneal injection of busulfan, followed by a single-dose administration of 1×10^5^ADSCs into the peritoneal cavity of these azoospermic mice, aiming to elucidate the *in vivo* mechanisms underlying its fertility-restoring effects in mice. The results showed that through actions of secreting different types of cell growth factors basic fibroblast growth factor/fibroblast growth factor 2 (FGF - 2), epidermal growth factor (EGF), and hepatocyte growth factor (HGF), etc., ADSCs could upregulate the mRNA levels of germ cell markers VASA homolog (VASA), deleted in azoospermia-like (DAZL), promyelocyic leukemia zinc finger (PLZF), Nanos homolog 3, Synaptonemal complex protein 3 (SCP3) and STELLA homolog (STELLA). These regulations further could improve the number of germ related cells in the seminiferous tubules of busulfan induced azoospermia mice, thereby significantly restoring the reproductive ability of busulfan induced azoospermia mice. This study provides an important cellular therapy basis for the treatment of male azoospermia (64). Furthermore, ROS, as one of the factors that affect male reproductive function, also play a key role in sperm agglutination and liquefaction processes (65). At an excessive concentration of ROS exceeding physiological levels, it may induce oxidative stress in testicular tissue and cause tissue damage, significantly compromising the sperm motility, capacitation process, acrosome response, and the ability to fuse with oocytes (66). Therefore, maintaining a steady-state ROS level is one of the critical strategies for preventing oxidative stress-induced testicular tissue damage. For example, supported by their ability of scavenging ROS, 1×10 (6) ADSCs injected as a single dose into the testes of azoospermia rabbits could significantly increase the level of glutathione (GSH) in testicular tissue and reduce the content of malondialdehyde (MDA) in cisplatin-induced azoospermia rabbit model, thereby enhancing the antioxidant capacity of rabbit testicular tissue and effectively preventing against cisplatin-induced oxidative stress damage (67). This study also confirmed the anti-apoptotic mechanism of ADSCs on testicular tissue cells, in which the occurrence of germ cell apoptosis in immature testes was a necessary physiological phenomenon under physiological conditions during the development of male germ cells. However, excessive activation of apoptosis can cause damage to the process of spermatogenesis and lead to reproductive dysfunction (68). The experiment further revealed that intratesticular injection of ADSCs significantly improved the excessive cell apoptosis in the seminiferous tubules of azoospermia rabbits through reducing the protein level of Caspase-3, an apoptosis marker, in testicular tissue cells, thereby restoring cisplatin-induced fertility in azoospermia rabbits (67).

In addition to improving chemotherapic agent-induced fertility decline, ADSCs also present with significant therapeutic potential in varicocele-induced male infertility. The impact of varicocele, a common disease that leads to male infertility, is mainly manifested as decreased sperm count, vitality, and reduced proportion of normal sperm morphology (69). Oxidative stress response caused by varicocele has been recognized to be one of the key factors affecting sperm quality parameters (70), clearly supporting the significance of inhibiting oxidative stress for treating male infertility caused by varicocele. Among them, superoxide dismutase (SOD), as a key antioxidant enzyme, can effectively eliminate free radicals and inhibit oxidative damage (71). Prior research revealed that a single-dose injection of 1×10 (6) ADSCs into the testes of mice with varicocele could effectively increase the level of SOD, while reducing the concentration of serum MDA, thereby effectively inhibiting oxidative stress response and improving varicocele-caused decline in sperm quality (72). Meanwhile, ADSCs can effectively improve sperm quality by inhibiting oxidative stress through multiple pathways. At the same time, Claudin-11 is a key tight junction protein expressed by Sertoli cells, which is also a major player in the formation and maintenance of the blood testis barrier (BTB) and is crucial for the process of spermatogenesis (73). The injection of ADSCs into the testes of mice with varicocele could not only regulate oxidative stress-related indicators, but also significantly increase the protein expression level of Claudin-11 in the testes of varicocele mice, further improving the BTB damage caused by varicocele, thereby restoring mouse fertility (74). Collectively, there are reasons to believe that ADSCs are promising for application in repairing testicular tissue and promoting normal sperm production through multiple mechanisms.

Testicular torsion is a common clinical emergency that may result in male infertility (75). Therefore, discussion on the main therapeutic mechanisms of ADSCs in treating testicular torsion may also provide valuable insights for their clinical application. According to existing research, reperfusion after testicular torsion could not only cause an increase in ROS production and oxidative damage, but also inhibit testicular endocrine function, subsequently disturbing the levels of reproductive hormones e.g., follicle stimulating hormone (FSH), luteinizing hormone (LH), and T, ultimately affecting sperm production (75). In the study of ADSCs restoring male infertility caused by testicular torsion, Hsiao et al. found that 3×10^4^ ADSCs could upregulate the protein expression of stem cell factor (SCF) in rats with testicular torsion, activate the SCF/cellular kinase in tyrosine (C-KIT) signaling pathway, significantly reduce the expression levels of Caspase-3 and Bax, thereby promoting germ cell proliferation and migration; it could also restore T secretion and improve sperm production in these rats (76).

In summary, single-dose injection of ADSCs at a specific quantity into different sites of different experimental subjects can effectively reconstruct the reproductive microenvironment. Its underlying mechanisms may involve the inhibition of oxidative stress response, reduction of testicular tissue cell apoptosis, promotion of BTB repair, regulation of reproductive hormone metabolism, and facilitation of the migration of reproductive cells. All these discoveries may provide innovative therapeutic regimes for various male infertility diseases.

### 3.2 Bone marrow mesenchymal stem cells

**Figure 3:**
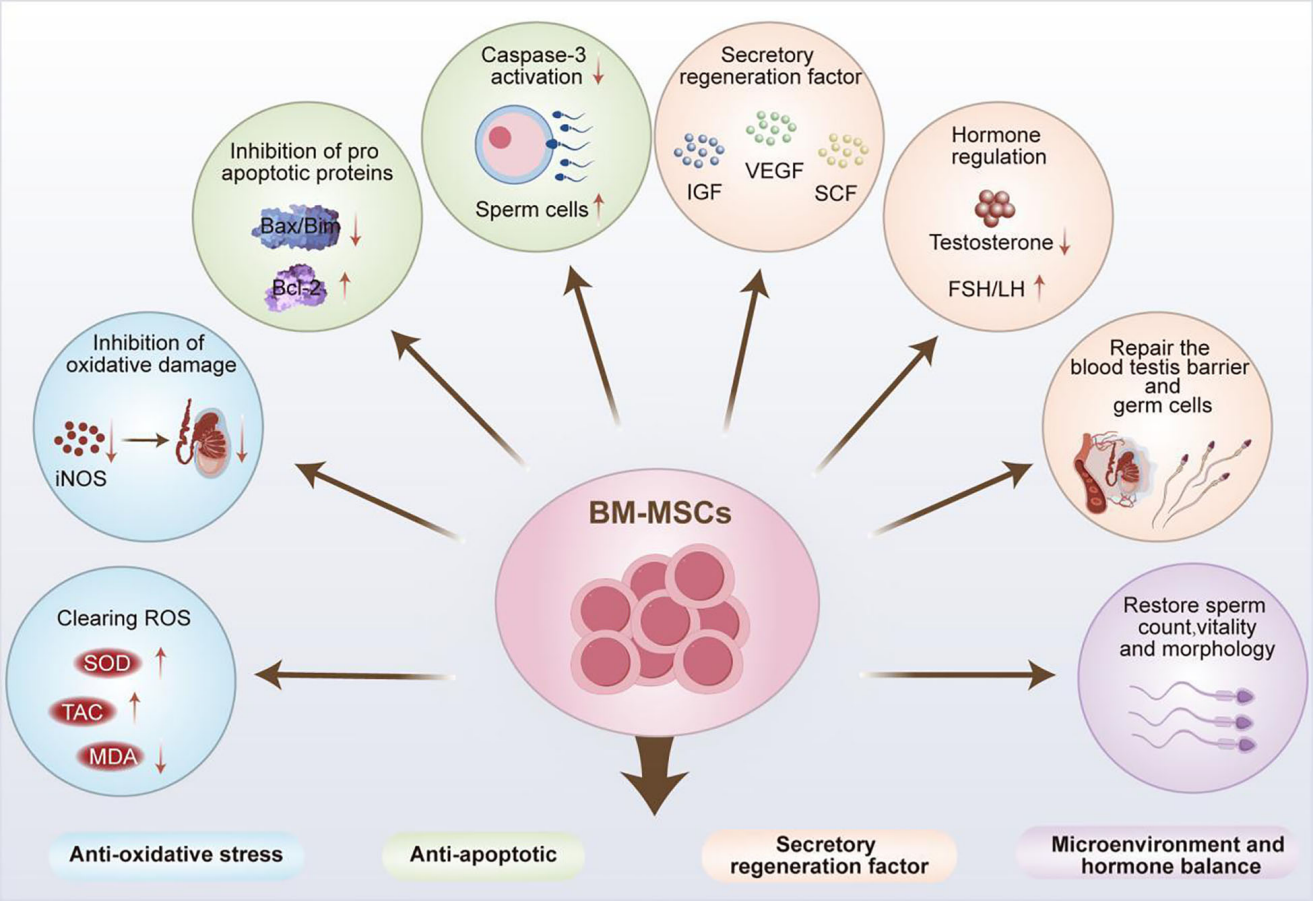
Application of BM-MSCs in the treatment of male infertility.

BM-MSCs have excellent tissue repair and immune regulatory properties, which hence have been widely used in the clinical treatment of various diseases (21). The application prospects of BM-MSCs in the field of male infertility treatment continue to expand as the study on their therapeutic mechanisms progressed, and they exhibit similar yet unique therapeutic characteristics as ADSCs. In addition to their significant therapeutic effect in male infertility caused by NOA and chemotherapic agents, significant advancements have been achieved in the treatment of male infertility induced by excessive apoptosis of testicular tissue cells and diabetes (**Figure 3**).

As a type of stem cells, MSCs also possess the multipotent differentiation potential of stem cells, and exhibit outstanding characteristics in the process of differentiation into germ cells. In a study of NOA treatment, Zhang et al. innovatively combined *in vitro* culture with *in vivo* differentiation. Rat BM-MSCs and testicular Sertoli cells were co-cultured in the Transwell system through simulating the reproductive microenvironment, which were then transplanted into the spermatogenic tubules of busulfan-induced azoospermia rats. There was improved differentiation efficiency of 1×10^5^ BM-MSCs-transplanted recipient tissues. The results showed significantly upregulated mRNA levels of various reproductive related markers e.g., VASA, STELLA, mothers against decapentaplegic homolog 1 (SMAD1), DAZL and the expression of spermatogenic related proteins e.g., germ cell nuclear factor (GCNF), heat shock protein 90 alpha (HSP90α), integrin β1, and C-KIT, without any adverse reactions. Therefore, BM-MSCs can effectively differentiate into germ cells *in vivo*, thereby promoting fertility recovery for the treatment of NOA (77).

In the research on male infertility caused by other tumor suppressor drugs, BM-MSCs have also demonstrated excellent therapeutic properties. Among them, cyclophosphamide (CYP) is a potent chemotherapic agent that plays an important role in tumor treatment (78). However, its anti-mitotic properties can hinder the development of testicular interstitial cells, affect the normal secretion of sex hormones, damage male reproductive function, and gradually lead to NOA (79). According to previous investigation, intraperitoneal injection of 1×10 (6) BM-MSCs in rats could significantly elevate SOD enzyme activity and total antioxidant capacity (TAC), thereby alleviating CYP-induced oxidative stress damage to testicular tissue in infertile rats. Moreover, this intervention could inhibit the elevation of serum FSH and LH caused by CYP by negatively regulating pituitary function, and restore the normal levels of FSH, LH, free T (FT) in the testes, and FT in the serum. Thus, BM-MSCs could manage the damage to testicular tissue and recover the disordered reproductive hormone levels caused by CYP in rats, thereby promoting spermatogenesis (79).

Furthermore, in a completed clinical trial on BM-MSCs (NCT02025270), 60 azoospermic patients with normal karyotypes were recruited and subjected to the injection of 20×10^6^ autologous BM-MSCs into their testes. During the follow-up one year later, 36 patients showed increased testicular volume, heightened T levels, and decreased LSH levels. Among these, sperm appeared in the semen of 3 men, sperm was found in 12 patients through puncture aspiration, and sperm was detected in 8 men via testicular biopsy. Despite a poor understanding of the specific therapeutic mechanism, BM-MSCs can be confirmed to exhibit intimate association with the regulation of sex hormone levels in the human body and restoration of normal spermatogenesis. Meanwhile, in another clinical trial (NCT02414295), congenital male oligozoospermia or azoospermia caused by Klinefelter syndrome was also investigated through the injection of BM-MSCs into the testes. Patients with oligozoospermia or azoospermia 3 – 12 months later were examined to measure the levels of sex hormones, azoospermia factors, testicular size, etc., thus identifying the therapeutic effect of BM-MSCs. However, no data of research has been retrieved so far, and its therapeutic effect remains to be further verified.

Furthermore, multiple experiments have verified the anti-excessive apoptosis effect of BM-MSCs on testicular tissue cells in the treatment of male infertility. Compared with the anti-apoptotic mechanism of ADSCs, BM-MSCs exhibit similar but more diverse anti-apoptotic pathways. For example, Zickri found that the injection of BM-MSCs into the testes could effectively reduce Caspase-3 expression in the spermatogenic epithelium of rats with busulfan-induced oligozoospermia. It could further significantly improved spermatogenic arrest, and reduced numbers of spermatogonia, spermatocytes, and sperm cells caused by increased cell apoptosis. Besides, the study also revealed a novel antioxidant mechanism of BM-MSCs. Specifically, BM-MSCs could significantly reduce the protein level ofinducible nitric oxide synthase (iNOS), an oxidative stress marker, in rat seminiferous epithelium, thereby alleviating oxidative stress damage to testicular tissue (80). Eventually, these findings support that MSCs can effectively restore normal sperm production by inhibiting oxidative stress and testicular tissue cell apoptosis through multiple pathways. Additional studies have also deciphered the unique mechanism of BM-MSCs in combating mitochondrial apoptosis in testicular tissue. They reported that BM-MSCs could inhibit the expression and mitochondria translocation of pro-apoptotic proteins B-cell lymphoma 2 (Bcl-2) interacting mediator of cell death (Bim) and Bcl-2-associated X protein (Bax). Meanwhile, BM-MSCs could upregulate anti-apoptotic Bcl-2 protein expression, prevent the release of Cytochrome C and apoptosis inducing factor (AIF) from mitochondria, activate Bim-Bax/Bcl-2 Cytochrome C-Caspase-dependent and Bim-Bax/Bcl-2 AIF-independent mitochondrial apoptosis pathways, thereby exerting a reparative effect on cadmium (Cd)-induced testicular injury (81).

At present, there is a continuous rise i n the incidence of diabetes with the change of lifestyle. The disease may induce male infertility by influencing the hypothalamus pituitary gonad (HPG) axis and inducing oxidative stress in reproductive organs (82). Therefore, hyperglycemia-related male infertility may be treatable by utilizing the antioxidant properties of MSCs and regulating the normal metabolism of sex hormonesy. As discovered by several researchers, intraperitoneal injection of BM-MSCs could significantly increase testicular TAC and reduce MDA level in diabetic rats, thereby mitigating oxidative stress damage, as well as improving sperm number, morphology, vitality and chromatin compression rate. Noticeably, co-culture of BM-MSCs with caffeine led to further enhanced antioxidant effect in the culture medium, providing a new potential therapy for the treatment of male infertility caused by diabetes (83).

In recent years, researchers have developed novel cultivation platforms to enhance the therapeutic efficacy of BM-MSCs. For example, Önen et al. developed a pumpless monodimethylsiloxane layered testis-on-chip platform, providing a continuous and stabilized microfluidic flow and real-time cellular paracrine contribution of allogeneic BM-MSCs. Compared with hanging droplets and non-BM-MSCs (controls), this platform could increase T concentration and improve the growth of seminiferous tubules for 42 days, revealing significant advantages in differentiating spermatogonia, VASA (+) total germ cells, meiotic cells (e.g., spermatocytes and sperm cells), and testicular maturation, thereby significantly improving *in vitro* sperm production (84).

Therefore, BM-MSCs can exert multiple functional mechanisms such as differentiating into germ cells *in vivo*, resisting oxidative stress and excessive apoptosis in testicular tissue, and improving spermatogenic ability to improve the spermatogenesis capacity. All these advantages can be utilized, coupled with the development of other new devices, to enhance its therapeutic efficacy, thereby providing a reliable reference for the treatment of male infertility.

### 3.3 Human umbilical cord mesenchymal stem cells

**Figure 4:**
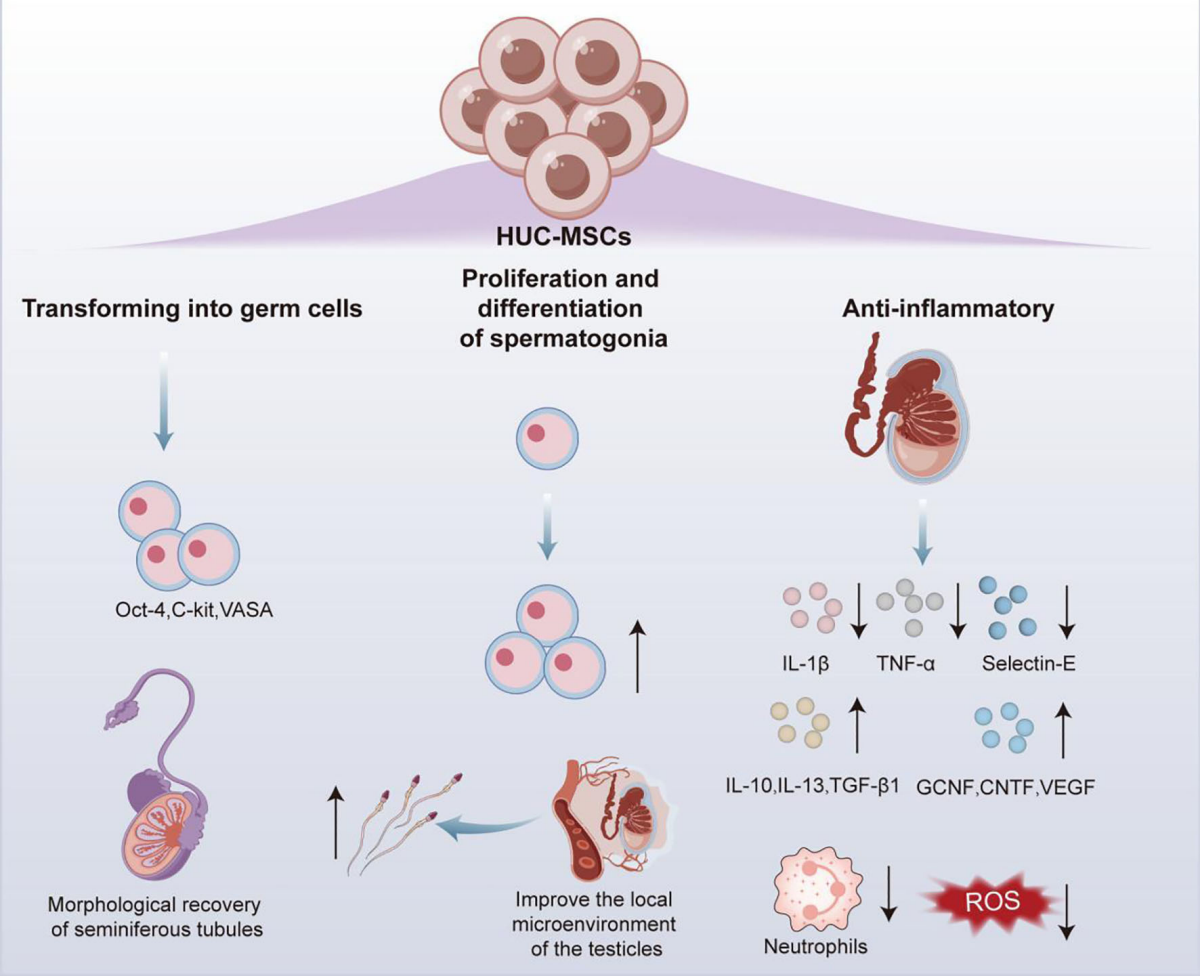
Application of HUC-MSCs in the treatment for male infertility.

HUC-MSCs have been demonstrated to enable the secretion of multiple cytokines and differentiation into different types of cells, while significantly reducing the risk of immune rejection and ethical controversies, supporting them an ideal source of cells in regenerative medicine field (85). Therefore, the relevant functional mechanisms of HUC-MSCs in testicular torsion as well as in *in vivo* and *in vitro* differentiation into germ cells were reported in studies on male infertility (**Figure 4**).

In male infertility caused by testicular torsion, administration of 10 (7) HUC-MSCs through tail vein injection was reported to effectively downregulate the mRNA expression levels of pro-inflammatory factors tumor necrosis factor alpha (TNF-α), IL - 1β and Selectin-E in the testes of rats with testicular torsion, while increasing the expression levels of anti-inflammatory cytokines (IL - 1ra, IL - 10, IL - 13, and TGF-β1) and the content of various nutritional cytokines germ cell nuclear factor (GCNF), ciliary neurotrophic factor, HGF, FGF, EGF, VEGF, etc. These changes significantly reduced neutrophil infiltration and ROS production, facilitating the alleviation of testicular torsion-induced inflammatory response and oxidative stress, and further promoting the proliferation and differentiation of spermatogonia (86). This discovery reveals the mechanism by which HUC-MSCs improve the local microenvironment of the testes through their anti-inflammatory properties to restore spermatogenesis, providing a novel theoretical basis of MSC therapy for male infertility.

In addition to its therapeutic properties, HUC-MSCs also exhibit the potential of *in vivo* and *in vitro* transforming into germ cells. As revealed by *in vivo* experiment, transplantation of HUC-MSCs into the seminiferous tubules of busulfan-induced germ cell deficient mice resulted in detectable expressions of germ cell-specific markers Octamer binding transcription factor 4 (Oct-4), α 6 integrin, C-KIT, and VASA in the mouse testes after 30 days, with sustained expressions for at least 120 days. Therefore, transplanted HUC-MSCs could survive for a long time and differentiate into germ cells in the testes of mice with normal immune function, while promoting the morphological recovery of damaged seminiferous tubules (85). Simultaneously, in *in vitro* investigation, when cell-free testicular tissue treated with mono-phosphoryl lipid A (MPLA) was co-cultured with RA-pretreated HUC-MSCs, there were obvious increase in the mRNA levels of migration related genes in HUC-MSCs C-X-C chemokine receptor type 4 (CXCR4), vascular cell adhesion molecule 1 (VCAM1), VEGF, Matrix Metalloproteinase 2 and very late antigen-4 (VLA4), and sperm differentiation marker related genes DAZL, VASA, and P-element induced wimpy testis like protein 2 (PIWIL2). It could be interpreted that HUC-MSCs cultured under specific niche conditions could effectively differentiate towards germ cells (87).

In summary, HUC-MSCs can also differentiate into germ cells *in vitro* and *in vivo*, beyond repairing testicular tissue damage through actions of anti-inflammatory and antioxidant stress, providing innovative strategies for the treatment of male infertility.

### 3.4 Other types of mesenchymal stem cells

Indeed, ADSCs, BM-MSCs, and HUC-MSCs have shown great potential in the treatment of male infertility caused by various etiologies, and they also exert their therapeutic effects through various pathways and molecular mechanisms. Other types of MSCs have also exhibit strong therapeutic potential, further enriching the application scope and theoretical basis of MSCs in treating male infertility (**Table 2**). In addition to unique therapeutic mechanism in the treatment of chemotherapeutic agent- and diabetes-induced male infertility, these other types of MSCs have also been confirmed to have effectiveness in the treatment of radiation-induced male infertility. These findings may provide an important theoretical basis and new research direction for clinical standardization of the selection of different types of MSCs for individualized treatment of male infertility.

**Table 2:** Research methods and therapeutic mechanisms of MSCs in the treatment for male infertility.

Therapeutic drug	Experimental subjects	Modeling method	SC source	Administration method	Dosage	Administration frequency	Research approach	Molecular mechanism	Therapeutic outcome	References
ADSCs	BALB/c mice	Intraperitoneal injection of busulfan	Human adipose tissue	Intraperitoneal injection	1×10^5^ ADSCs	Single dose injection	Cytokines that promote cell growth, Nutrient factor generation	mRNA expression of Testicular tissue: ↑VASA, ↑DAZL, ↑PLZF, ↑Nanos homolog 3, ↑SCP3, and ↑STELLA	Differentiation into germ cells	(64)
	New Zealand rabbits	Intratesticular injection of cisplatin	Adipose tissue in the scapular region of rabbits	Intratesticular injection	1×10^6^ ADSCs	Single dose injection	Anti-oxidative stress, Anti apoptosis, regulating hormone secretion	Protein expression of Testicular tissue: ↓ROS, ↓MDA, ↑GSH, and Cytoplasm of Spermatogonia: ↓Caspase-3	Differentiation into germ cells	(67)
	Wistar rats	Varicocelectomy	Human adipose tissue	Intratesticular injection	Per 1cc containing 1.0×_6_ Cells	Single dose injection	Anti-oxidative stress	Testicular tissue:↑SOD, and ↓MDA	Improvement of sperm quality	(72)
	Wistar rats	Varicocelectomy	Adipose tissue in the inguinal region of albino mice	Intratesticular injection	50µL ADSCs-CM 30G	Twice a week, lasting for 4 weeks	Anti-oxidative stress, Tissue repair	Protein expression of Serum:↑SOD, ↓MDA, and Testicular tissue:↑Claudin-11	Improvement of sperm quality	(74)
	SD rats	Testicular torsion surgery	Human orbital adipose tissue	Intratesticular injection	3×10^4^ ADSCs	Single dose injection	Anti-oxidative stress, anti-apoptosis, Hormone level regulation	Proteinn expression of Testicular tissue: ↑SOD, ↓MDA, ↓Caspase-3, ↓Bax, and Restore T secretion	Improvement of sperm quality	(88)
	Human ADSCs	RA co-culture	Human adipose tissue	ADSCs and RA culture *in vitro*	-	-	*In vitro* differentiation toward germ cells	mRNA expression of Testicular tissue: ↑Oct4, ↑PIWILl2, ↑SSEA-1, ↑STRA8, and protein expression of ↑Oct-4	Differentiation into germ cells	(89)
	Syrian hamsters	Intraperitoneal injection of busulfan	Hamster abdominal and cervical adipose tissue	Intratesticular injection	10^6^ ADSCs	Single dose injection	Repair damage to reproductive organs	-	Repair testicular tissue	(90)
	Wistar rats	-	-	Co culture of Bisphenol A and Rat Epididymid Sperm	50 µg, 100 µg	4 hours before bisphenol A intervention, injection twice	Relieve mitochondrial damage, Anti-oxidative stress	Testicular tissue mitochondrion: ↓ROS, ↑SOD, and ↑CAT	Improvement of sperm quality	(91)
	Wistar rats	Intraperitoneal injection of busulfa	Rat Peritoneal adipose tissue	Intratesticular injection	10^6^ ADSCs	Single dose injection	Differentiation into germ cells *in vivo*	Testicular tissue: ↑VASA, and ↑SCP3 Number of positive protein staining results	Differentiation into germ cells	(92)
HUC-MSCs	Kunming mice	-	Human umbilical cord tissue	Testicular tissue extracted primary cells and HUC-MSCs culture *in vitro*	-	-	*In vitro* differentiation into germ cells	mRNA expression of Testicular tissue: ↑Oct4, ↑C-KIT, ↑Integrin α6, ↑STELLA, and ↑VASA	Differentiation into germ cells	(93)
	SD rats	Testicular torsion surgery	Human umbilical cord tissue	Tail vein injection	10^7^ HUC -MSCs	Single dose injection	Anti-inflammatory, Anti-oxidative stress	mRNA expression of Testicular tissue: ↑IL-1ra, ↑IL-10, ↑IL-13, ↓TNF-α, ↓IL-1, and ↓ROS	Repairtesticular tissue, spermatogenesis improvement	(86)
	Kunming mice	Intraperitoneal injection of busulfan	Human umbilical cord tissue	Intratesticular injection	2.5×10^5^ HUC -MSCs	Single dose injection	*In vivo* differentiation into germ cells	Testicular tissue: ↑Oct-4, ↑α6 Integrin, ↑C-KIT, and ↑VASA	Differentiation into germ cells	(85)
	Human fetus testicular tissue		Human umbilical cord tissue	Humhan fetus testicular tissue and HUC-MSCs co-culture *in vitro*	-	-	*In vitro* Differentiation into germ cells	mRNA expression of Testicular tissue: ↑C-KIT, ↑VASA, and ↑SSEA-4	Differentiation into germ cells	(94)
	BALB/c mice	Intraperitoneal injection of busulfan	Human umbilical cord tissue	Intratesticular injection	About 1×10^5^ HUC -MSCs	Single dose injection	*In vivo* differentiation into germ cells	mRNA expression of Testicular tissue: ↑VASA, ↑DAZL, ↑PIWIL1, ↑Testis Nuclear Protein 2, ↑Phosphoglycerate Kinase 2, ↑SCP3, and ↑STRA8	Improvement of spermatogenesis	(95)
	sheep testicular tissue	-	Human umbilical cord tissue	MPLA and RA processed testicular tissueand HUC-MSCs culture *in vitro*	-	-	*In vitro* Differentiation into germ cells	mRNA expression of Testicular tissue: ↑CXCR4, ↑VCAM1, ↑VEGF, ↑MMP2, ↑VLA4, ↑DAZL, ↑DDX4, and ↑PIWIL2	Differentiation into germ cells	(87)
	Sertoli cells in mice	-	Human umbilical cord tissue	Sertoli Cell Conditional culture medium and HUC-MSCs culture *in vitro*	-	-	*In vitro* differentiation into germ cells	mRNA expression of Testicular tissue: ↑Oct-4, ↑Nanog, ↑PLZF, ↑STRA8, and ↑Protamine 1	Differentiation into germ cells	(96)
BM-MSCs	SD rats	Intraperitoneal injection busulfan	Rat myeloid tissue	Intratubular injection of spermatogenic tubules	10^5^ BM-MSCs	Single dose injection	Differentiation into germ cells	mRNA expression of Testicular tissue: ↑VASA, ↑STELLA, and ↑DAZL, Protein expression of ↑GCNF, ↑HSP90α↑integrin β1, and ↑C-KIT,	Differentiation into germ cells	(77)
	SD rats	Intraperitoneal injection of CYP	Rat myeloid tissue	Intratesticular injection	1×10^6^ BM-MSCs	Every 3 weeks	Anti-oxidative stress, Hormone level regulation	Testicular tissue: ↑SOD enzyme activity, ↑TAC, serum: ↓FSH, and ↓LH	Improvement of spermatogenesis	(79)
	Albino rats	Intraperitoneal injection of busulfan	Rat myeloid tissue	Intratesticular injection	2.5*10^6^ BM-MSC/per testicle	Single dose injection	Anti-oxidative stress, anti-apoptosis	Protein expression level of Testicular tissue: ↓Caspase-3, ↓iNOS	Improvement of spermatogenesis	(80)
	Wistar rats	Intraperitoneal injection of Niacinamide and Streptozotocin	Rat myeloid tissue	Intraperitoneal injection	-	Single dose injection	Anti-oxidative stress	Testicular tissue: ↑TAC, and ↓MDA	Improvement of sperm quality	(83)
	Wistar rats	Intraperitoneal injection of CdCl2	Rat myeloid tissue	Retroorbital injection	1×10^7^ BM-MSCs	Two injections in consecutive days	Anti-apoptosis	Protein expression of Testicular tissue: ↓Bim, ↓Bax protein expression, and ↑Bcl-2	Repair testicular tissue	(81)
	Wistar rats	Testicular torsion surgery	Rat myeloid tissue	Intraperitoneal injection	1ml BM-MSCs conditioned medium	Single dose injection	Anti-inflammatory	Testicular tissue: ↓ Neutrophil recruitment	Improvement of sperm quality	(97)
	Wistar rats	Intraperitoneal injection of adriamycin	Rat myeloid tissue	Tail vein injection	2×10^6^ BM-MSCs	Single dose injection	Anti-oxidative stress	Testicular tissue: ↑TAC, and ↓MDA	Repair testicular tissue	(98)
HAM-MSCs	Wistar rats	X ray irradiation	Human full-term placenta	Intratesticular injection	2.5×10^5^ HAMSCs centrifuged cells	Single dose injection	Anti-apoptosis, Hormone regulation	Testicular tissue: ↓Capase12, ↓Capase3, serum: ↓FSH, ↑LH, and ↑T	Improvement of spermatogenesis	(99)
	C57BL/6 mice	Intraperitoneal injection of busulfan	-	Intraperitoneal injection	1×10^7^ HAMSC	Single dose injection	Anti-oxidative stress, anti-apoptosis, Hormone regulation	Protein expression of Testicular tissue: ↑GR, ↑SOD, ↑GPX , ↑CAT, ↑T, ↑Bcl-2, ↑Survivin, ↓Capase3↓, and Capase9	Improvement of spermatogenesis	(100)
HP-MSCs	C57BL/6 mice	Intraintestinal injection of busulfan	Human placenta sample	Intratesticular injection	5×10^6^ HPMSCs	Single dose injection	Regulating sperm autophagy	Protein expression and mRNA level of Testicular tissue: ↓p62, and ↓LC3	Improvement of spermatogenesis	(101)
BR-MSCs	SD rats	Intraperitoneal injection of streptozotocin	Human breast milk samples	Intraperitoneal injection	2×10^7^ Br-MSCs	Two injections, with a 14 day interval	Hormone regulation	Serum: ↑FSH, ↑LH, and ↑T	Improvement of spermatogenesis	(102)

Human amniotic MSCs (HAM MSCs) exhibit high proliferation potential, low immunogenicity, low inflammatory response, and non-invasive culture advantages, emerging as an additional SC therapy gradually (103). Cetinkaya et al. found that HAC MSCs could inhibit the activation of inositol requiring enzyme 1α by reducing the expression of glucose regulated protein 78, effectively reversing endoplasmic reticulum stress (ER) in testicular tissue, reducing the production of apoptosis factors (e.g., Capase12 and Capase3) in rat seminiferous tubules caused by ER stress, eventually exerting an effect on mitigating radiation-induced testicular tissue damage in rat; moreover, HAM MSCs could effectively inhibit radiation-induced elevation of serum FSH and LH, improve the resultant decrease in T content, further contributing to the normal spermatogenesis (99). In another study, HAM MSCs were discovered to effectively inhibit the production of ROS, lactate dehydrogenase, and MDA, while increasing the levels of glutathione reductase (GR), SOD, glutathione peroxidase, and CAT, thereby improving testicular tissue damage caused by oxidative stress in mice. Through upregulating the mRNA levels of germ cell specific genes (e.g., DAZL, VASA, and PIWIL2), as well as meiotic genes (e.g., SCP3, Cyclin A1, and STRA8), HAM MSCs could increase protein expressions of survivin and Bcl-2, while reducing those of Capase3 and Capase9, thereby enhancing the anti-apoptotic ability of testicular tissue cells in mice, thus treating busulfan-induced damage to testicular tissues in mice (100).

Recently, great concern has been attached to the therapeutic potential of human placental MSCs (HP MSCs), especially their pivotal role in regulating sperm autophagy (104–106). Autophagy, has been recognized as an important intracellular metabolic regulatory mechanism, which is essential in maintaining cellular homeostasis (107). With the deepening of research on male infertility, the key role of sperm autophagy in spermatogenesis and reproductive processes is becoming increasingly prominent, and its excessive activation or inhibition can significantly affect male reproductive function (108). HP MSCs have been found to have the ability of effectively reducing the protein and mRNA levels of autophagy-related markers Sequetosome 1 (p62) and microtubule-associated protein 1 light chain 3 (LC3) in busulfan-induced mouse testicular toxicity model, while improving the significant decrease in sperm autophagy caused by busulfan, thereby boosting the repair of mouse reproductive function (101).

In addition, some researchers also reported the unique therapeutic mechanism of human breast milk MSCs (BR MSCs) in diabetes-induced male infertility. According to relevant research results, BR MSCs could enhance insulin level by interacting with insulin receptors, thereby enhancing energy metabolism in kisspeptin neurons, stimulating HPG axis, effectively restoring the serum levels of FSH, LH and total T of diabetic rats, thereby improving diabetes-induced fertility decline (102).

## 4 The application of MSC-Exos in male infertility

EVs are a group of heterogeneously sized, cell-derived nanoparticles operating as proficient mediators of intercellular communication, which are well-established players in various physiological and pathological processes by transporting bioactive molecules to target cells, providing novel therapeutic strategies for disease treatment (109). Noticeably, besides inheriting the powerful immune regulatory and tissue regenerative properties of mother cell MSCs in terms of therapeutic characteristics, MSC Exos can also effectively avoid adverse reactions (e.g., infusion-related toxicity) that may occur during the implementation of MSC therapies, thus having significant therapeutic advantages (11). The potential application of MSC Exos has been a hotspot in the treatment of male infertility in recent decades. Study on its therapeutic mechanisms underlying the treatment of male infertility caused by chemotherapic agents and environmental pollution may contribute to the establishment of a new feasible approach to address male infertility.

Guo et al. co-cultured bone marrow MSC-derived Exos (BMSC Exos) with CYP-induced testicular tissues in mice. Corresponding results showed that based on the uptake of BMSC Exos, mouse spermatogonia significantly reduced the intracellular phosphorylation levels of extracellular signal regulated kinase (ERK), AKT, and p38 Mitogen activated protein kinase (p38MAPK) proteins. As a result, such phosphorylation reduction inhibited the activation of p38MAPK/ERK and AKT signaling pathways, promoted the repair of damaged testicular tissue, and effectively treated CYP-induced testicular tissue damage (110). Current evidence has also confirmed that human umbilical cord MSC-derived Exos (HUCMSC Exos) can significantly reduce the levels of ROS in mouse testicular tissues, inhibit oxidative stress response, downregulate the expression of pro-apoptotic proteins Bax and caspase-3, upregulate the protein level of cell proliferation marker Bcl-2, significantly increase the mRNA expression of mouse reproductive related markers (e.g., DAZL, STRA8, and VASA), as well as connexin 43 intercellular adhesion molecule-1, cytoskeleton related genes β-catenin and androgen receptor in testicular tissue, thereby effectively promoting the proliferation and migration of mouse spermatogonia (111).

In the study of testicular injury caused by environmental pollution, MSC Exos also exhibit preferable therapeutic properties. For instance, induced pluripotent SC-derived MSC-derived Exos (iMSC Exos) could significantly increase the expression of GC1 spermatogonia (spg) cells (GC1-SPG), and reduce the number of lesions with H2AX+p53 binding protein 1+ by inhibiting the protein level of phosphorylated gamma-histone H2A. X (γ H2AX), thereby alleviating DNA damage and improving Cd-induced decline in mouse spermatogenic ability. Meanwhile, iMSC Exos could effectively increase the formation of autophagosomes and autolysosomes in mouse germ cell specific protein 1/spermatogenesis associated protein, enhance autophagy flux and reduce P62 protein accumulation, improve Cd-induced autophagy flux deficiency, promote spermatogonial proliferation and differentiation, protect mice from Cd-induced germ cell apoptosis, and cope with the decline in reproductive ability caused by Cd in environmental pollution (112).

Indeed, bacterial infections of the reproductive organs and tract are regarded as one of the important causes among numerous factors contributing to male infertility. Among them, *Chlamydia trachomatis* infection is a common sexually transmitted disease that has been supported by a large body of evidence recently to play a role in sperm dysfunction and poor sperm quality (113). For example, *Chlamydia trachomatis* infection can induce sperm apoptosis by increasing sperm mitochondrial membrane potential and activating Caspase-3, thereby leading to male infertility (114). However, *Chlamydia trachomatis* infection is usually asymptomatic, and the screening cost is relatively high, posing great challenge to its screening. Conventional clinical treatments, such as oral azithromycin, can effectively inhibit *Chlamydia trachomatis* infection, but they may impair the body’s ability to maintain a sustained protective immune response against Chlamydia, increasing the difficulty of treatment after reinfection. Therefore, there is an urgent need to develop novel therapeutic approaches to address the damage to *Chlamydia trachomatis* infection-induced male reproductive capacity. It has been reported that through the analysis of the proven therapeutic properties of MSC-Exos in antibacterial, anti-infective, and antioxidant effects from previous experiments, MSC-Exos may have potential therapeutic value for male infertility induced by *Chlamydia trachomatis* infection (115). However, there are currently no specific experimental results to support this argument, and the feasibility of this inference still needs further verification.

In addition, MSC Exos have shown promising application prospects in sperm storage. Drug therapy-induced gonadal toxicity has been accepted as a major contributor to male infertility for many years (116). Semen cryopreservation through traditional method encounter with several problems such as DNA damage and decreased vitality of sperm after thawing, ultimately reducing the fertilization potential of frozen-thawed sperm (117). However, male patients undergoing drug therapies can only use the sperm with significantly reduced fertilization potential after freeze-thaw cycles. Therefore, a key approach in response to this challenge may be the effective preservation of sperm or spermatogonial SCs. It has been reported that MSC-secreted micorvesicles could effectively enhance the TAC of frozen-thawed sperm, while increasing the expression of adhesion molecules CD54, CD106, CD29, and CD44 on the surface of sperm, improving the adhesion of frozen-thawed sperm, eventually significantly improving the survival and motility of frozen-preserved sperm (117). It can be acknowledged that numerous studies have confirmed the therapeutic potential of EVs in improving sperm quality and fertilization ability (118–120). However, further research is needed to verify whether EVs in MSCs do indeed play a critical role in sperm storage.

Therefore, by inheriting the therapeutic properties of MSCs in treating male infertility, such as antioxidant effects and anti-apoptosis of testicular tissue cells, MSC-Exos can be used to treat the damage to reproductive organs caused by the use of chemotherapeutic drugs and post-chemotherapeutic drugs. Moreover, MSC-Exos show great potential in addressing the damage to male reproductive organs and tracts caused by bacterial infections, as well as in sperm storage (**Table 3**).

**Table 3:** The application of MSC-Exos in male infertility.

Therapeutic drug	Experimental subjects	Modeling method	SC source	Administration method	Dosage	Administration frequency	Research approach	Molecular mechanism	Therapeutic outcome	References
BMSC-Exos	SD rats	Intraperitoneal injection of CYP	Isolationof exosomes in BM-MSCs	Tail vein injection	100 μg for 7 days continuously	Single-dose injection	Anti-apoptosis	Inactivation of p38MAPK/ERK and AKT signaling pathways	Repair of testicular tissue	(110)
HUCMSC-Exos	ICR mice	Treatment with busulfan after the separation of GC - 1 spermatogonia	Isolationof exosomes in HUCMSC-Exos	*In vitro* culture	-	-	Antioxidant stress and anti-apoptosis	Testicular tissues: Protein expression of ↓ ROS, ↓ Bax, ↓ caspase-3, and ↑ Bcl-2	Repair of testicular tissue	(111)
iMSC-Exos	C57BL/6 mice	Oral cadmium chloride for 4 weeks	Isolationof exosomes in MSCs	Intratesticular injection	20μg for 4 weeks continuously	Single-dose injection	Anti-apoptosis and regulation of autophagy	Testicular tissues: ↑ Bcl-2 protein expression, ↓ γH2AX protein level, and ↓ p62 protein accumulation	Repair of testicular tissue	(112)

## 5 Challenges in the use of MSCs

At present, the clinical translation of MSCs, despite well-documented safety profile, still faces many constraints, including ethical risks, immune rejection reactions, tumorigenicity, as well as the scarcity and complexity of applications. Therefore, there are a limited number of SC therapies that have been clinically validated and successfully applied (121–123). Tumorigenicity is believed to be the most severe challenge among the many uncertain factors of MSCs. As revealed by recent investigation (124), MSCs could promote the growth of tumor blood vessels and recruit immunosuppressive myeloid cells by interacting directly with tumor cells and interacting with other components of the tumor microenvironment, thereby driving tumor immune rejection aas well as playing a key role in tumor progression and metastasis. Furthermore, compared to healthy tissue-derived MSCs, tumor tissue-retained MSCs were found to exhibit unique expression profiles and significant tumor-promoting properties (125).

In addition, the clinical application of MSCs has been restricted greatly owing to the disadvantages of high preparation cost, complex preparation technology, and relatively underdeveloped preparation system, making it difficult to achieve mass production of products and relative uniformity in quality. Taking BM-MSCs as an example, there is an extremely small number of BM-MSCs that can be extracted from the bone marrow. Their preparation involves multiple steps such as *in vitro* isolation, culture, differentiation, and preservation, each of which requires professional operations by technicians as well as the use of a large number of professional and expensive consumables and equipment. After the preparation of qualified products, they also face risks of stem cell senescence after expansion and tumorigenesis due to mutation, resulting in their extremely limited application in experiments or clinical settings, thereby compromising their further large-scale application (126).

Despite safer and more reliable therapeutic properties compared to MSCs, MSC Exos may still be accompanied by potential tumor risks, high preparation costs, and incomplete standardization systems, which limit their further clinical applications (127, 128). For example, while potentially inhibiting tumor growth by regulating specific signaling pathways, MSC Exos might also promote tumor growth, angiogenesis, and metastasis by delivering specific miRNAs (e.g., miRNA-21 and miRNA-34a) and proteins. This dual effect may have an intimate association with factors such as cultivation conditions and tissue sources, underscoring the urgent need for further standardized research to clarify its functional mechanism and optimize its application strategies (129).

Meanwhile, even stricter conditions are proposed for the preparation of MSC-Exos. Firstly, in terms of quality control, the lack of standardized protocols for exosome isolation and purification triggers evident variations in product characteristics and therapeutic efficacy. Although ultracentrifugation has been acccepted as the “gold standard” for exosome isolation, several issues remain to be addressed, such as expensive equipment, long processing time (4 – 6 hours), limited recovery rate, and purity problems caused by co-precipitation of other extracellular vesicles and protein complexes (127). Furthermore, researchers have devoted much of their energy in significantly increasing exosome yields in industrial production through methods such as genetic engineering, parent cell pretreatment, 3D culture, and optimization of culture materials, while also reducing batch differences and contamination risks (130). Nevertheless, these techniques may alter the biological functions of stem cell exosomes in ways that are not yet fully understood, which may pose potential risks to some extent. Therefore, it may be difficult to predict the actual effects of these methods in large-scale production and clinical applications.

In the aspect of research on male infertility treatment, MSCs have made significant achievements in animal experiments, tyet with the absence of relevant human clinical trials due to various factors. Although experiments have shown that human fetal testicular tissue can effectively differentiate into male germ cells when co-cultured with HUC-MSCs *in vitro* (94), it should be noted that there are still significant differences between fetal testicular tissue and adult male testicular tissue. Moreover, whether such tissue, when directly implanted into the testes of adult males via injection or other methods, can exert the same therapeutic potential in the human body without other side effects remains to be verified. In addition, owing to suboptimal technical level currently, little is known about the specific molecular mechanism of MSCs restoring normal sperm production. Related studies often attribute this phenomenon to a specific therapeutic characteristic of MSCs, restricting the confirmation of its precise intervention mechanism. As a result, related research is highly uncertain and subjective, which is not conducive to the in-depth development of subsequent systematic research.

Simultaneously, ethical risks are also issues that need to be emphasized squarely in the application of MSCs for treating male infertility. Therefore, how to establish a correct concept to view the new assisted reproductive models, whether in the clinical trial stage or the application stage, remains an ideological prerequisite affecting their further development.

## 6 Summary and prospect

The present study reviews the latest research progress in the application of MSCs and MSC Exos in the treatment of male infertility induced by external factors, such as chemotherapeutic agent use, diabetes, environmental pollution, and research findings about their abilities of *in vivo* and *in vitro* differentiation into germ cells under specific conditions (**Table 2**). This study systematically analyzes the therapeutic characteristics of MSCs from different sources (e.g., ADSCs, BM-MSCs, HU-MSCs, etc.) and their application mechanisms in the treatment of male infertility, which exhibit significant antioxidant stress, anti-apoptosis, immune regulation, and tissue repair properties. They can effectively repair damage to testicular tissues caused by various etiologies, rebuild the spermatogenic microenvironment, and restore normal sperm production. Significantly, MSCs can promote the proliferation and differentiation into germ cells through the activation of multiple signaling pathways by releasing various growth factors, cytokines, and chemokines. In addition, MSC-Exos, as an important mediator for MSCs to exert therapeutic effects, can both inherit the therapeutic characteristics of MSCs, and avoid potential adverse reactions effectively during the application of MSC therapies, providing a new therapeutic option for male infertility.

Indeed, MSCs have shown broad application prospects in male infertility treatment, but the presence of many challenges may hinder their further clinical translation. The potential tumorigenicity and immune rejection risks of MSCs need to be addressed through stricter safety assessments and quality control systems. Furthermore, the actual clinical application of MSC Exos may be impeded by their high preparation cost and incomplete standardization system, although they exhibit safer and more reliable therapeutic properties compared to MSCs. Noticeably, available data generated from existing research are mostly based on rodent models, with a lack of data from human clinical trial, necessitating further verification on the therapeutic efficacy and safety of MSCs in humans. Simultaneously, further research on the underlying mechanisms is also required given that there is currently a poor understanding of the specific molecular mechanism by which MSCs restore normal sperm production.

In response to the above challenges, future research should focus on the establishment of standardized processes for the preparation of MSCs and MSC Exos to ensure their quality stability and clinical application safety; efforts should also be made to explore more efficient and relatively low-cost large-scale production models for MSCs and MSC-Exos; in-depth exploration of the molecular mechanisms underlying the repair of damage to testicular tissues by MSCs and MSC Exos, particularly a systematic identification of key regulatory molecules and signaling pathways through high-throughput sequencing technology and multi-omics analysis; performance of more preclinical studies and early clinical trials to verify the effectiveness and safety of MSCs in treating male infertility; investigation on the combined use of MSCs with existing therapies (e.g., drug therapy, surgical treatment, or assisted reproductive technology) to further improve treatment efficacy; as well as the development of. individualized treatment plans for MSCs targeting specific etiologies to meet the clinical needs of different patients.

With the development of biomaterials science, microfluidic technology, and tissue engineering, the construction of MSC-based tissue-engineered testes will also become a research hotspot in the future. It is expected to achieve a complete spermatogenesis process *in vitro* through the construction of tissue-engineered testes with biomimetic structures, which may be a source of sperm for patients with severe oligospermia or azoospermia. Moreover, the application of gene editing technologies such as CRISPR-Cas9 may enable a targeted modification of MSCs to enhance their specific therapeutic functions, which will further enhance the effectiveness of MSCs in treating male infertility.

In conclusion, the emerging MSCs and MSC Exos have shown great potential in the field of male infertility treatment. By addressing existing technological bottlenecks, delving into their functional mechanisms, and conducting standardized clinical trials, MSC therapies are anticipated to become an innovative solution for male infertility, bringing new hope for patients with refractory male infertility. However, to ensure the reliability and safety of MSCs’ therapeutic effects, the translation from basic research to clinical application still requires collaborative efforts from multiple disciplines and centers.

## References

[1] Calogero AE Cannarella R Agarwal A Hamoda TAA Rambhatla A Saleh R. The renaissance of male infertility management in the golden age of andrology. World J Mens Health. (2023) 41:237–54. doi: 10.5534/wjmh.220213, PMID: 36649928 PMC10042649

[2] Arora M Mehta P Sethi S Anifandis G Samara M Singh R. Genetic etiological spectrum of sperm morphological abnormalities. J Assisted Reprod Genet. (2024) 41:2877–929. doi: 10.1007/s10815-024-03274-8, PMID: 39417902 PMC11621285

[3] Calvert JK Fendereski K Ghaed M Bearelly P Patel DP Hotaling JM. The male infertility evaluation still matters in the era of high efficacy assisted reproductive technology. Fertility Sterility. (2022) 118:34–46. doi: 10.1016/j.fertnstert.2022.05.008, PMID: 35725120

[4] Sharma A Minhas S Dhillo WS Jayasena CN. Male infertility due to testicular disorders. J Clin Endocrinol Metab. (2021) 106:E442–E59. doi: 10.1210/clinem/dgaa781, PMID: 33295608 PMC7823320

[5] Qin Y Ge GR Yang P Wang LL Qiao YS Pan GQ. An update on adipose-derived stem cells for regenerative medicine: where challenge meets opportunity. Advanced Sci. (2023) 10:e2207334. doi: 10.1002/advs.202207334, PMID: 37162248 PMC10369252

[6] Hoang DM Pham PT Bach TQ Ngo ATL Nguyen QT Phan TTK. Stem cell-based therapy for human diseases. Signal Transduction Targeted Ther. (2022) 7:272. doi: 10.1038/s41392-022-01134-4, PMID: 35933430 PMC9357075

[7] Shan YL Zhang MY Tao EX Wang J Wei N Lu Y. Pharmacokinetic characteristics of mesenchymal stem cells in translational challenges. Signal Transduction Targeted Ther. (2024) 9:242. doi: 10.1038/s41392-024-01936-8, PMID: 39271680 PMC11399464

[8] Feng B Feng XD Yu YD Xu HY Ye QQ Hu RT. Mesenchymal stem cells shift the pro-inflammatory phenotype of neutrophils to ameliorate acute lung injury. Stem Cell Res Ther. (2023) 14:197. doi: 10.1186/s13287-023-03438-w, PMID: 37553691 PMC10408228

[9] Yan JX Liang JJ Cao YX El Akkawi MM Liao X Chen XJ. Efficacy of topical and systemic transplantation of mesenchymal stem cells in a rat model of diabetic ischemic wounds. Stem Cell Res Ther. (2021) 12:220. doi: 10.1186/s13287-021-02288-8, PMID: 33789742 PMC8010295

[10] Zhidu S Ying T Rui J Chao Z. Translational potential of mesenchymal stem cells in regenerative therapies for human diseases: challenges and opportunities. Stem Cell Res Ther. (2024) 15:266. doi: 10.1186/s13287-024-03885-z, PMID: 39183341 PMC11346273

[11] Lotfy A AboQuella NM Wang HJ. Mesenchymal stromal/stem cell (MSC)-derived exosomes in clinical trials. Stem Cell Res Ther. (2023) 14:66. doi: 10.1186/s13287-023-03287-7, PMID: 37024925 PMC10079493

[12] Onen S Kose S Yersal N Korkusuz P. Mesenchymal stem cells promote spermatogonial stem/progenitor cell pool and spermatogenesis in neonatal mice *in vitro* . Sci Rep. (2022) 12:11494. doi: 10.1038/s41598-022-15358-5, PMID: 35798781 PMC9263145

[13] Luo YX Xie LL Mohsin A Ahmed W Xu CZ Peng Y. Efficient generation of male germ-like cells derived during co-culturing of adipose-derived mesenchymal stem cells with Sertoli cells under retinoic acid and testosterone induction. Stem Cell Res Ther. (2019) 10:91. doi: 10.1186/s13287-019-1181-5, PMID: 30867048 PMC6415496

[14] Kangari P Talaei-Khozani T Razeghian-Jahromi I Razmkhah M. Mesenchymal stem cells: amazing remedies for bone and cartilage defects. Stem Cell Res Ther. (2020) 11:492. doi: 10.1186/s13287-020-02001-1, PMID: 33225992 PMC7681994

[15] Lan TX Luo M Wei XW. Mesenchymal stem/stromal cells in cancer therapy. J Hematol Oncol. (2021) 14:195. doi: 10.1186/s13045-021-01208-w, PMID: 34789315 PMC8596342

[16] Li ZT Wang YM Wang H Wang HY Shang YX Wang SH. Self-assembled DNA composite-engineered mesenchymal stem cells for improved skin-wound repair. Small. (2024) 20:e2310241. doi: 10.1002/smll.202310241, PMID: 38441385

[17] Neo SH Her Z Othman R Tee CA Ong L Wang YH. Expansion of human bone marrow-derived mesenchymal stromal cells with enhanced immunomodulatory properties. Stem Cell Res Ther. (2023) 14:259. doi: 10.1186/s13287-023-03481-7, PMID: 37726837 PMC10510228

[18] Gonçalves CES da Silva RO Hastreiter AA Vivian GK Makiyama EN Borelli P. Reduced protein intake and aging affects the sustainment of hematopoiesis by impairing bone marrow mesenchymal stem cells. J Nutr Biochem. (2024) 124:109511. doi: 10.1016/j.jnutbio.2023.109511, PMID: 37913969

[19] Shi Y Wang S Zhang WW Zhu YH Fan ZQ Huang YS. Bone marrow mesenchymal stem cells facilitate diabetic wound healing through the restoration of epidermal cell autophagy via the HIF - 1α/TGF-β1/SMAD pathway. Stem Cell Res Ther. (2022) 13:314. doi: 10.1186/s13287-022-02996-9, PMID: 35841007 PMC9284495

[20] Mazini L Rochette L Amine M Malka G. Regenerative capacity of adipose derived stem cells (ADSCs), comparison with mesenchymal stem cells (MSCs). Int J Mol Sci. (2019) 20:2523. doi: 10.3390/ijms20102523, PMID: 31121953 PMC6566837

[21] Chu DT Phuong TNT Tien NLB Tran DK Thanh VV Quang TL. An update on the progress of isolation, culture, storage, and clinical application of human bone marrow mesenchymal stem/stromal cells. Int J Mol Sci. (2020) 21:708. doi: 10.3390/ijms21030708, PMID: 31973182 PMC7037097

[22] Ko EH Yoon T Lee YJ Kim J Park YB. ADSC secretome constrains NK cell activity by attenuating IL - 2-mediated JAK-STAT and AKT signaling pathway via upregulation of CIS and DUSP4. Stem Cell Res Ther. (2023) 14:329. doi: 10.1186/s13287-023-03516-z, PMID: 37964351 PMC10648656

[23] Li LY Li JC Guan HF Oishi H Takahashi S Zhang C. Human umbilical cord mesenchymal stem cells in diabetes mellitus and its complications: applications and research advances. Int J Med Sci. (2023) 20:1492–507. doi: 10.7150/ijms.87472, PMID: 37790847 PMC10542192

[24] Mebarki M Abadie C Larghero J Cras A. Human umbilical cord-derived mesenchymal stem/stromal cells: a promising candidate for the development of advanced therapy medicinal products. Stem Cell Res Ther. (2021) 12:152. doi: 10.1186/s13287-021-02222-y, PMID: 33637125 PMC7907784

[25] Jin HJ Bae YK Kim M Kwon SJ Jeon HB Choi SJ. Comparative analysis of human mesenchymal stem cells from bone marrow, adipose tissue, and umbilical cord blood as sources of cell therapy. Int J Mol Sci. (2013) 14:17986–8001. doi: 10.3390/ijms140917986, PMID: 24005862 PMC3794764

[26] Xiao K Liu C Wang HM Hou F Shi YH Qian ZR. Umbilical cord mesenchymal stem cells overexpressing CXCR7 facilitate treatment of ARDS-associated pulmonary fibrosis via inhibition of Notch/Jag1 mediated by the Wnt/β-catenin pathway. Biomedicine Pharmacotherapy. (2023) 165:115124. doi: 10.1016/j.biopha.2023.115124, PMID: 37454589

[27] Li T Zhou L Fan MQ Chen ZX Yan L Lu HS. Human umbilical cord-derived mesenchymal stem cells ameliorate skin aging of nude mice through autophagy-mediated anti-senescent mechanism. Stem Cell Rev Rep. (2022) 18:2088–103. doi: 10.1007/s12015-022-10418-9, PMID: 35864432

[28] Luo YL Qu JY He ZY Zhang MH Zou ZW Li LC. Human umbilical cord mesenchymal stem cells improve the status of hypoxic/ischemic cerebral palsy rats by downregulating nogoA/ngR/rho pathway. Cell Transplant. (2023) 32:9636897231210069. doi: 10.1177/09636897231210069, PMID: 37982384 PMC10664427

[29] Jiang XK Luo XY Cai CH Bai YQ Ding H Yue H. Umbilical cord mesenchymal stem cells in ulcerative colitis treatment: efficacy and possible mechanisms. Stem Cell Res Ther. (2024) 15:272. doi: 10.1186/s13287-024-03878-y, PMID: 39218946 PMC11368034

[30] Feng H Liu Q Deng ZY Li H Zhang HJ Song JY. Human umbilical cord mesenchymal stem cells ameliorate erectile dysfunction in rats with diabetes mellitus through the attenuation of ferroptosis. Stem Cell Res Ther. (2022) 13:450. doi: 10.1186/s13287-022-03147-w, PMID: 36064453 PMC9444126

[31] Song J Liu JD Cui C Hu HQ Zang N Yang MM. Mesenchymal stromal cells ameliorate diabetes-induced muscle atrophy through exosomes by enhancing AMPK/ULK1-mediated autophagy. J Cachexia Sarcopenia Muscle. (2023) 14:915–29. doi: 10.1002/jcsm.13177, PMID: 36708027 PMC10067482

[32] Xiang E Han B Zhang Q Rao W Wang ZF Chang C. Human umbilical cord-derived mesenchymal stem cells prevent the progression of early diabetic nephropathy through inhibiting inflammation and fibrosis. Stem Cell Res Ther. (2020) 11:336. doi: 10.1186/s13287-020-01852-y, PMID: 32746936 PMC7397631

[33] Mildmay-White A Khan W. Cell surface markers on adipose-derived stem cells: A systematic review. Curr Stem Cell Res Ther. (2017) 12:484–92. doi: 10.2174/1574888X11666160429122133, PMID: 27133085

[34] Ghazanfari R Zacharaki D Li HZ Lim HC Soneji S Scheding S. Human primary bone marrow mesenchymal stromal cells and their *in vitro* progenies display distinct transcriptional profile signatures. Sci Rep. (2017) 7:10338. doi: 10.1038/s41598-017-09449-x, PMID: 28871088 PMC5583257

[35] Kacham S Bhure TS Eswaramoorthy SD Naik G Rath SN Parcha SR. Human umbilical cord-derived mesenchymal stem cells promote corneal epithelial repair *in vitro* . Cells. (2021) 10. doi: 10.3390/cells10051254, PMID: 34069578 PMC8160941

[36] Kang SY Yasuhara R Tokumasu R Funatsu T Mishima K. Adipose-derived mesenchymal stem cells promote salivary duct regeneration via a paracrine effect. J Oral Biosciences. (2023) 65:104–10. doi: 10.1016/j.job.2023.01.006, PMID: 36736698

[37] Kawada-Horitani E Kita S Okita T Nakamura Y Nishida H Honma Y. Human adipose-derived mesenchymal stem cells prevent type 1 diabetes induced by immune checkpoint blockade. Diabetologia. (2022) 65:1185–97. doi: 10.1007/s00125-022-05708-3, PMID: 35511238 PMC9174328

[38] Lee TL Lai TC Lin SR Lin SW Chen YC Pu CM. Conditioned medium from adipose-derived stem cells attenuates ischemia/reperfusion-induced cardiac injury through the microRNA-221/222/PUMA/ETS-1 pathway. Theranostics. (2021) 11:3131–49. doi: 10.7150/thno.52677, PMID: 33537078 PMC7847683

[39] Lv MZ Zhang SM Jiang B Cao SR Dong YQ Cao L. Adipose-derived stem cells regulate metabolic homeostasis and delay aging by promoting mitophagy. FASEB J. (2021) 35:e21709. doi: 10.1096/fj.202100332R, PMID: 34143518

[40] Dong B Wang C Zhang J Zhang JR Gu YN Guo XP. Exosomes from human umbilical cord mesenchymal stem cells attenuate the inflammation of severe steroid-resistant asthma by reshaping macrophage polarization. Stem Cell Res Ther. (2021) 12:e21709. doi: 10.1186/s13287-021-02244-6, PMID: 33761997 PMC7988945

[41] Li M Li J Wang Y Jiang GC Jiang HG Li MD. Umbilical cord-derived mesenchymal stem cells preferentially modulate macrophages to alleviate pulmonary fibrosis. Stem Cell Res Ther. (2024) 15:475. doi: 10.1186/s13287-024-04091-7, PMID: 39696548 PMC11657361

[42] Shi JL Yao H Chong H Hu X Yang J Dai XM. Tissue-engineered collagen matrix loaded with rat adipose-derived stem cells/human amniotic mesenchymal stem cells for rotator cuff tendon-bone repair. Int J Biol Macromolecules. (2024) 282:137144. doi: 10.1016/j.ijbiomac.2024.137144, PMID: 39488324

[43] Kadono M Nakashima A Ishiuchi N Sasaki K Miura Y Maeda S. Adipose-derived mesenchymal stem cells cultured in serum-free medium attenuate acute contrast-induced nephropathy by exerting anti-apoptotic effects. Stem Cell Res Ther. (2023) 14:337. doi: 10.1186/s13287-023-03553-8, PMID: 37993965 PMC10664307

[44] Liu HF Huang HC Liu YF Yang YX Deng HC Wang XM. Adipose-derived mesenchymal stem cells inhibit hepatic stellate cells activation to alleviate liver fibrosis via Hippo pathway. Stem Cell Res Ther. (2024) 15:378. doi: 10.1186/s13287-024-03988-7, PMID: 39449061 PMC11515333

[45] Xin JF Zhou L Zhang L Guo K Yang DY. Neuroprotective effects of human adipose-derived mesenchymal stem cells in oxygen-induced retinopathy. Cell Transplantation. (2023) 32:9636897231213309. doi: 10.1177/09636897231213309, PMID: 38018498 PMC10687918

[46] Li SY Sun JC Yang JX Yang Y Ding HF Yu BY. Gelatin methacryloyl (GelMA) loaded with concentrated hypoxic pretreated adipose-derived mesenchymal stem cells(ADSCs) conditioned medium promotes wound healing and vascular regeneration in aged skin. Biomaterials Res. (2023) 27:11. doi: 10.1186/s40824-023-00352-3, PMID: 36782342 PMC9926638

[47] Chen L Luo W Wang YZ Song XB Li SL Wu J. Directional homing of glycosylation-modified bone marrow mesenchymal stem cells for bone defect repair. J Nanobiotechnology. (2021) 19:228. doi: 10.1186/s12951-021-00969-3, PMID: 34332597 PMC8325817

[48] Li J Lv Y Wang HY Liu Y Ren JX Wang HP. Cardiomyocyte-like cell differentiation by FGF - 2 transfection and induction of rat bone marrow mesenchymal stem cells. Tissue Cell. (2021) 73:101665. doi: 10.1016/j.tice.2021.101665, PMID: 34695652

[49] Qin ZY Han YX Du YF Zhang YX Bian YF Wang RY. Bioactive materials from berberine-treated human bone marrow mesenchymal stem cells promote alveolar bone regeneration by regulating macrophage polarization. Sci China-Life Sci. (2024) 67:1010–26. doi: 10.1007/s11427-023-2454-9, PMID: 38489007

[50] Wu L Rong C Zhou Q Zhao X Zhuansun XM Wan S. Bone Marrow Mesenchymal Stem Cells Ameliorate Cisplatin-Induced Renal Fibrosis *via* miR-146a-5p/Tfdp2 Axis in Renal Tubular Epithelial Cells. Front Immunol. (2021) 11. doi: 10.3389/fimmu.2020.623693, PMID: 33664736 PMC7921314

[51] Li J Li HYZ Cai SM Bai S Cai HB Zhang XM. CD157 in bone marrow mesenchymal stem cells mediates mitochondrial production and transfer to improve neuronal apoptosis and functional recovery after spinal cord injury. Stem Cell Res Ther. (2021) 12:289. doi: 10.1186/s13287-021-02305-w, PMID: 34001228 PMC8127190

[52] Chen P Yuan MQ Yao LC Xiong ZY Liu PJ Wang Z. Human umbilical cord-derived mesenchymal stem cells ameliorate liver fibrosis by improving mitochondrial function via Slc25a47-Sirt3 signaling pathway. Biomedicine Pharmacotherapy. (2024) 171:116133. doi: 10.1016/j.biopha.2024.116133, PMID: 38198960

[53] Liao Y Fu ZQ Huang YF Wu SD Wang Z Ye ST. Interleukin-18-primed human umbilical cord-mesenchymal stem cells achieve superior therapeutic efficacy for severe viral pneumonia via enhancing T-cell immunosuppression. Cell Death Dis. (2023) 14:66. doi: 10.1038/s41419-023-05597-3, PMID: 36707501 PMC9883134

[54] Sun ZQ Gu P Xu HJ Zhao W Zhou YJ Zhou LY. Human umbilical cord mesenchymal stem cells improve locomotor function in parkinson’s disease mouse model through regulating intestinal microorganisms. Front Cell Dev Biol. (2022) 9. doi: 10.3389/fcell.2021.808905, PMID: 35127723 PMC8810651

[55] Zhang H Wang XS Hu B Li PC Abuduaini Y Zhao HM. Human umbilical cord mesenchymal stem cells attenuate diabetic nephropathy through the IGF1R-CHK2-p53 signalling axis in male rats with type 2 diabetes mellitus. J Zhejiang University-Science B. (2024) 25:568–80. doi: 10.1631/jzus.B2300182, PMID: 39011677 PMC11254681

[56] Chiu YH Liang YH Hwang JJ Wang HS. IL - 1β stimulated human umbilical cord mesenchymal stem cells ameliorate rheumatoid arthritis via inducing apoptosis of fibroblast-like synoviocytes. Sci Rep. (2023) 13:15344. doi: 10.1038/s41598-023-42585-1, PMID: 37714911 PMC10504325

[57] Dong L Li XY Leng WY Guo ZK Cai TY Ji X. Adipose stem cells in tissue regeneration and repair: From bench to bedside. Regenerative Ther. (2023) 24:547–60. doi: 10.1016/j.reth.2023.09.014, PMID: 37854632 PMC10579872

[58] Huang YJ Zhu MD Liu Z Hu RA Li F Song YF. Bone marrow mesenchymal stem cells in premature ovarian failure: Mechanisms and prospects (vol 13, 997808, 2022). Front Immunol. (2022) 13. doi: 10.3389/fimmu.2022.997808, PMID: 36389844 PMC9646528

[59] Bongso A Fong CY. The therapeutic potential, challenges and future clinical directions of stem cells from the wharton’s jelly of the human umbilical cord. Stem Cell Rev Rep. (2013) 9:226–40. doi: 10.1007/s12015-012-9418-z, PMID: 23233233

[60] Bunnell BA. Adipose tissue-derived mesenchymal stem cells. Cells. (2021) 10:3433. doi: 10.3390/cells10123433, PMID: 34943941 PMC8700397

[61] Huang R Xia H Lin W Wang Z Li L Deng J. Riluzole reverses blood-testis barrier loss to rescue chemotherapy-induced male infertility by binding to TRPC. Cells. (2024) 13:2016. doi: 10.3390/cells13232016, PMID: 39682764 PMC11640501

[62] Zhao LJ Zhao J Dong ZH Xu SY Wang D. Mechanisms underlying impaired spermatogenic function in orchitis induced by busulfan. Reprod Toxicology. (2023) 115:1–7. doi: 10.1016/j.reprotox.2022.11.002, PMID: 36372306

[63] Park HJ Kim JS Lee R Song H. Cisplatin induces apoptosis in mouse neonatal testes organ culture. Int J Mol Sci. (2022) 23:13360. doi: 10.3390/ijms232113360, PMID: 36362147 PMC9658841

[64] Ganjibakhsh M Mehraein F Koruji M Bashiri Z. The therapeutic potential of adipose tissue-derived mesenchymal stromal cells in the treatment of busulfan-induced azoospermic mice. J Assisted Reprod Genet. (2022) 39:153–63. doi: 10.1007/s10815-021-02309-8, PMID: 34519944 PMC8866597

[65] Hamada A Sharma R du Plessis SS Willard B Yadav SP Sabanegh E. Two-dimensional differential in-gel electrophoresis-based proteomics of male gametes in relation to oxidative stress. Fertility Sterility. (2013) 99:1216. doi: 10.1016/j.fertnstert.2012.11.046, PMID: 23312230

[66] Dutta S Sengupta P Slama P Roychoudhury S. Oxidative stress, testicular inflammatory pathways, and male reproduction. Int J Mol Sci. (2021) 22:10043. doi: 10.3390/ijms221810043, PMID: 34576205 PMC8471715

[67] Ismail HY Shaker NA Hussein S Tohamy A Fathi M Rizk H. Cisplatin-induced azoospermia and testicular damage ameliorated by adipose-derived mesenchymal stem cells. Biol Res. (2023) 56:2. doi: 10.1186/s40659-022-00410-5, PMID: 36653814 PMC9850593

[68] Qian Y-C Xie Y-X Wang C-S Shi Z-M Jiang C-F Tang Y-Y. Mkrn2 deficiency induces teratozoospermia and male infertility through p53/PERP-mediated apoptosis in testis. Asian J Andrology. (2020) 22:414. doi: 10.4103/aja.aja_76_19, PMID: 31489847 PMC7406093

[69] Cannarella R Shah R Hamoda T Boitrelle F Saleh R Gul M. Does varicocele repair improve conventional semen parameters? A meta-analytic study of before-after data. World J Mens Health. (2024) 42:92–132. doi: 10.5534/wjmh.230034, PMID: 37382284 PMC10782123

[70] Hassani-Bafrani H Najaran H Razi M Rashtbari H. Berberine ameliorates experimental varicocele-induced damages at testis and sperm levels; evidences for oxidative stress and inflammation. Andrologia. (2019) 51:e13179. doi: 10.1111/and.13179, PMID: 30334274

[71] Rhodes MA Carraway MS Piantadosi CA Reynolds CM Cherry AD Wester TE. Carbon monoxide, skeletal muscle oxidative stress, and mitochondrial biogenesis in humans. Am J Physiology-Heart Circulatory Physiol. (2009) 297:H392–H9. doi: 10.1152/ajpheart.00164.2009, PMID: 19465554 PMC2711725

[72] Siregar S Noegroho BS Adriansjah R Mustafa A Bonar A. The effect of intratesticular injection of human adipose-derived mesenchymal cell on testicular oxidative stress and spermatogenesis process in the varicocele rat model. Res Rep Urology. (2021) 13:759–65. doi: 10.2147/RRU.S330634, PMID: 34676179 PMC8519792

[73] Miao CJ Wang ZJ Wang X Huang WY Gao X Cao Z. Deoxynivalenol Induces Blood-Testis Barrier Dysfunction through Disrupting p38 Signaling Pathway-Mediated Tight Junction Protein Expression and Distribution in Mice. J Agric Food Chem. (2023) 71:12829–38. doi: 10.1021/acs.jafc.3c03552, PMID: 37590035

[74] Serefoglu EC Kolbasi B Bulbul MV Karabulut S Cakici C Ozdemir RZG. Therapeutic effects of mesenchymal stem cell conditioned medium in rat varicocele model. World J Mens Health. (2024) 43:437447. doi: 10.5534/wjmh.240059, PMID: 39344116 PMC11937358

[75] Jacobsen FM Rudlang TM Fode M Ostergren PB Sonksen J Ohl DA. The impact of testicular torsion on testicular function. World J Mens Health. (2020) 38:298–307. doi: 10.5534/wjmh.190037, PMID: 31081295 PMC7308234

[76] Hsiao CH Ji ATQ Chang CC Cheng CJ Lee LM Ho JHC. Local injection of mesenchymal stem cells protects testicular torsion-induced germ cell injury. Stem Cell Res Ther. (2015) 6:113. doi: 10.1186/s13287-015-0079-0, PMID: 26025454 PMC4449584

[77] Zhang DY Liu X Peng JP He DW Lin T Zhu J. Potential spermatogenesis recovery with bone marrow mesenchymal stem cells in an azoospermic rat model. Int J Mol Sci. (2014) 15:13151–65. doi: 10.3390/ijms150813151, PMID: 25062349 PMC4159785

[78] Izdebska M Zielinska W Krajewski A Halas-Wisniewska M Mikolajczyk K Gagat M. Downregulation of MMP - 9 enhances the anti-migratory effect of cyclophosphamide in MDA-MB-231 and MCF - 7 breast cancer cell lines. Int J Mol Sci. (2021) 22:12783. doi: 10.3390/ijms222312783, PMID: 34884588 PMC8657655

[79] Ibrahim D Abozied N Maboud SA Alzamami A Alturki NA Jaremko M. Therapeutic potential of bone marrow mesenchymal stem cells in cyclophosphamide-induced infertility. Front Pharmacol. (2023) 14. doi: 10.3389/fphar.2023.1122175, PMID: 37033609 PMC10073512

[80] Zickri MB Moustafa MH Fasseh AEE Kamar SS. Antioxidant and antiapoptotic paracrine effects of mesenchymal stem cells on spermatogenic arrest in oligospermia rat model. Ann Anatomy-Anatomischer Anzeiger. (2021) 237:151750. doi: 10.1016/j.aanat.2021.151750, PMID: 33940119

[81] Wang YJ Yan J Zou XL Guo KJ Zhao Y Meng CY. Bone marrow mesenchymal stem cells repair cadmium-induced rat testis injury by inhibiting mitochondrial apoptosis. Chemico-Biological Interactions. (2017) 271:39–47. doi: 10.1016/j.cbi.2017.04.024, PMID: 28457857

[82] Zhu XB Niu ZH Fan WM Cheng MY Chen Q Zhang AJ. Alternative polarization of resident macrophages improves hyperglycemia-associated male infertility. Iscience. (2022) 25:104430. doi: 10.1016/j.isci.2022.104430, PMID: 35669521 PMC9162941

[83] Goshadezehn P Babaei-Balderlou F Razi M Najafi GR Abtahi-Foroushani M. A caffeine pre-treatment and sole effect of bone-marrow mesenchymal stem cells-derived conditioned media on hyperglycemia-suppressed fertilization. Biomedicine Pharmacotherapy. (2023) 165:115130. doi: 10.1016/j.biopha.2023.115130, PMID: 37413898

[84] Önen S Atik AC Gizer M Köse S Yaman Ö Külah H. A pumpless monolayer microfluidic device based on mesenchymal stem cell-conditioned medium promotes neonatal mouse *in vitro* spermatogenesis. Stem Cell Res Ther. (2023) 14:127. doi: 10.1186/s13287-023-03356-x, PMID: 37170113 PMC10173473

[85] Chen H Tang Q-L Wu X-Y Xie L-C Lin L-M Ho G-Y. Differentiation of human umbilical cord mesenchymal stem cells into germ-like cells in mouse seminiferous tubules. Mol Med Rep. (2015) 12:819–28. doi: 10.3892/mmr.2015.3528, PMID: 25815600 PMC4438948

[86] Zhong L Yang MB Zou XY Du T Xu HM Sun J. Human umbilical cord multipotent mesenchymal stromal cells alleviate acute ischemia-reperfusion injury of spermatogenic cells via reducing inflammatory response and oxidative stress. Stem Cell Res Ther. (2020) 11:294. doi: 10.1186/s13287-020-01813-5, PMID: 32680554 PMC7366899

[87] Shojaeian A Mehri-Ghahfarrokhi A Banitalebi-Dehkordi M. Monophosphoryl lipid A and retinoic acid combinations increased germ cell differentiation markers expression in human umbilical cord-derived mesenchymal stromal cells in an *in vitro* ovine acellular testis scaffold. Int J Mol Cell Med. (2020) 9:288–95. doi: 10.22088/IJMCM.BUMS.9.4.288, PMID: 33688486 PMC7936076

[88] Hsiao C Ji A Chang C Cheng C Lee L Ho J. LOCAL INJECTION OF MESENCHYMAL STEM CELLS PROTECTS TESTICULAR TORSION-INDUCED GERM CELL INJURY. Cytotherapy. (2016) 18:S71–S. doi: 10.1016/j.jcyt.2016.03.239, PMID: 26025454 PMC4449584

[89] Liu HH Chen MT Liu LL Ren SS Cheng PP Zhang H. Induction of human adipose-derived mesenchymal stem cells into germ lineage using retinoic acid. Cell Reprogramming. (2018) 20:127–34. doi: 10.1089/cell.2017.0063, PMID: 29620445

[90] Karimaghai N Tamadon A Rahmanifar F Mehrabani D Jahromi AR Zare S. Spermatogenesis after transplantation of adipose tissue-derived mesenchymal stem cells in busulfan-induced azoospermic hamster. Iranian J Basic Med Sci. (2018) 21:660–7. doi: 10.22038/IJBMS.2018.29040.7010, PMID: 30140403 PMC6098960

[91] Soleimani MZ Khorsandi L Asadi-Fard Y Rezaei-Tazangi F Ashtari A. Protective effects of adipose mesenchymal stem cell secretome on oxidative stress-induced bisphenol-A in isolated rat testes mitochondria and sperm quality. JBRA assisted reproduction. (2025) 29:53–60. doi: 10.5935/1518-0557.20240089, PMID: 39688440 PMC11867245

[92] Cakici C Buyrukcu B Duruksu G Haliloglu AH Aksoy A Isik A. Recovery of fertility in azoospermia rats after injection of adipose-tissue-derived mesenchymal stem cells: the sperm generation. BioMed Res Int. (2013) 2013:529589. doi: 10.1155/2013/529589, PMID: 23509736 PMC3590610

[93] Huang P Lin LM Wu XY Tang QL Feng XY Lin GY. Differentiation of human umbilical cord wharton’s jelly-derived mesenchymal stem cells into germ-like cells *in vitro* . J Cell Biochem. (2010) 109:747–54. doi: 10.1002/jcb.22453, PMID: 20052672

[94] Amidi F Ataie Nejad N Agha Hoseini M Nayernia K Mazaheri Z Yamini N. *In vitro* differentiation process of human Wharton’s jelly mesenchymal stem cells to male germ cells in the presence of gonadal and non-gonadal conditioned media with retinoic acid. In Vitro Cell Dev Biol Anim. (2015) 51:1093–101. doi: 10.1007/s11626-015-9929-4, PMID: 26427713

[95] Yang R-F Liu T-H Zhao K Xiong C-L. Enhancement of mouse germ cell-associated genes expression by injection of human umbilical cord mesenchymal stem cells into the testis of chemical-induced azoospermic mice. Asian J Andrology. (2014) 16:698–704. doi: 10.4103/1008-682X.129209, PMID: 24830694 PMC4215652

[96] Maghami RG Mirzapour T Bayrami A. Differentiation of mesenchymal stem cells to germ-like cells under induction of Sertoli cell-conditioned medium and retinoic acid. Andrologia. (2018) 50:e12287. doi: 10.1111/and.12887, PMID: 28944567

[97] Sharifian P Yari S Hasanein P Nezhad YM. Conditioned medium of bone marrow mesenchymal stem cells improves sperm parameters and reduces histological alteration in rat testicular ischaemia/reperfusion model. Andrologia. (2022) 54:e14624. doi: 10.1111/and.14624, PMID: 36270637

[98] Abdelaziz MH El-Din EYS El-Dakdoky MH Ahmed TA. The impact of mesenchymal stem cells on doxorubicin-induced testicular toxicity and progeny outcome of male prepubertal rats. Birth Defects Res. (2019) 111:906–19. doi: 10.1002/bdr2.1535, PMID: 31210400

[99] Cetinkaya-Un B Un B Akpolat M Andic F Yazir Y. Human amnion membrane-derived mesenchymal stem cells and conditioned medium can ameliorate X-irradiation-induced testicular injury by reducing endoplasmic reticulum stress and apoptosis. Reprod Sci. (2022) 29:944–54. doi: 10.1007/s43032-021-00753-6, PMID: 34642916

[100] Qian CF Meng QX Lu JF Zhang LY Li H Huang BX. Human amnion mesenchymal stem cells restore spermatogenesis in mice with busulfan-induced testis toxicity by inhibiting apoptosis and oxidative stress. Stem Cell Res Ther. (2020) 11:290. doi: 10.1186/s13287-020-01803-7, PMID: 32678012 PMC7367397

[101] Lu J Liu Z Shu M Zhang L Xia W Tang L. Human placental mesenchymal stem cells ameliorate chemotherapy-induced damage in the testis by reducing apoptosis/oxidative stress and promoting autophagy. Stem Cell Res Ther. (2021) 12:199. doi: 10.1186/s13287-021-02275-z, PMID: 33743823 PMC7981860

[102] Khamis T Abdelalim AF Abdallah SH Saeed AA Edress NM Arisha AH. Early intervention with breast milk mesenchymal stem cells attenuates the development of diabetic-induced testicular dysfunction via hypothalamic Kisspeptin/Kiss1r-GnRH/GnIH system in male rats. Biochim Et Biophys Acta-Molecular Basis Disease. (2020) 1866:165577. doi: 10.1016/j.bbadis.2019.165577, PMID: 31672553

[103] Huang BX Ding CY Zou QY Lu JF Wang W Li H. Human amniotic fluid mesenchymal stem cells improve ovarian function during physiological aging by resisting DNA damage. Front Pharmacol. (2020) 11. doi: 10.3389/fphar.2020.00272, PMID: 32273842 PMC7113373

[104] Yao QG Chen WY Yu YD Gao FQ Zhou JH Wu J. Human placental mesenchymal stem cells relieve primary sclerosing cholangitis via upregulation of TGR5 in mdr2-/- mice and human intrahepatic cholangiocyte organoid models. Research. (2023) 6:0207. doi: 10.34133/research.0207, PMID: 37600495 PMC10433880

[105] Xue LL Du RL Bi N Xiao QX Sun YF Niu RZ. Transplantation of human placental chorionic plate-derived mesenchymal stem cells for repair of neurological damage in neonatal hypoxic-ischemic encephalopathy. Neural Regeneration Res. (2024) 19:2027–35. doi: 10.4103/1673-5374.390952, PMID: 38227532 PMC11040304

[106] de Laorden EH Simón D Milla S Portela-Lomba M Mellén M Sierra J. Human placenta-derived mesenchymal stem cells stimulate neuronal regeneration by promoting axon growth and restoring neuronal activity. Front Cell Dev Biol. (2023) 11. doi: 10.3389/fcell.2023.1328261, PMID: 38188022 PMC10766706

[107] Liu S Yao S Yang H Liu S Wang Y. Autophagy: Regulator of cell death. Cell Death Disease. (2023) 14:648. doi: 10.1038/s41419-023-06154-8, PMID: 37794028 PMC10551038

[108] Wang M Zeng L Su P Ma L Zhang M Zhang YZ. Autophagy: a multifaceted player in the fate of sperm. Hum Reprod Update. (2022) 28:200–31. doi: 10.1093/humupd/dmab043, PMID: 34967891 PMC8889000

[109] Rezaie J Feghhi M Etemadi T. A review on exosomes application in clinical trials: perspective, questions, and challenges. Cell Communication Signaling. (2022) 20:145. doi: 10.1186/s12964-022-00959-4, PMID: 36123730 PMC9483361

[110] Guo XB Zhai JW Xia H Yang JK Zhou JH Guo WB. Protective effect of bone marrow mesenchymal stem cell-derived exosomes against the reproductive toxicity of cyclophosphamide is associated with the p38MAPK/ERK and AKT signaling pathways. Asian J Andrology. (2021) 23:386–91. doi: 10.4103/aja.aja_98_20, PMID: 33565424 PMC8269825

[111] Yue DZ Wang F Han Y Xiong CL Yang RF. Exosomes derived from umbilical cord mesenchymal stem cells ameliorate male infertility caused by busulfan *in vivo* and in *vitro* . Ecotoxicology Environ Safety. (2024) 272:116063. doi: 10.1016/j.ecoenv.2024.116063, PMID: 38306818

[112] Chen ZH Mo JH Yang QY Guo ZX Li XY Xie DM. MSC-derived exosomes mitigate cadmium-induced male reproductive injury by ameliorating DNA damage and autophagic flux. Ecotoxicology Environ Saf. (2024) 276:116306. doi: 10.1016/j.ecoenv.2024.116306, PMID: 38631218

[113] Keikha M Hosseininasab-Nodoushan SA Sahebkar A. Association between chlamydia trachomatis infection and male infertility: A systematic review and meta-analysis. Mini Rev Med Chem. (2023) 23:746–55. doi: 10.2174/1389557522666220827160659, PMID: 36043714

[114] Sellami H Znazen A Sellami A Mnif H Louati N Ben Zarrouk S. Molecular detection of Chlamydia trachomatis and other sexually transmitted bacteria in semen of male partners of infertile couples in Tunisia: the effect on semen parameters and spermatozoa apoptosis markers. PloS One. (2014) 9:e98903. doi: 10.1371/journal.pone.0098903, PMID: 25019616 PMC4096407

[115] Izadi M Dehghan Marvast L Rezvani ME Zohrabi M Aliabadi A Mousavi SA. Mesenchymal stem-cell derived exosome therapy as a potential future approach for treatment of male infertility caused by chlamydia infection. Front Microbiol. (2021) 12:785622. doi: 10.3389/fmicb.2021.785622, PMID: 35095800 PMC8792933

[116] Shetty G Mitchell JM Lam TNA Phan TT Zhang J Tailor RC. Postpubertal spermatogonial stem cell transplantation restores functional sperm production in rhesus monkeys irradiated before and after puberty. Andrology. (2021) 9:1603–16. doi: 10.1111/andr.13033, PMID: 33960147 PMC8815151

[117] Mokarizadeh A Rezvanfar MA Dorostkar K Abdollahi M. Mesenchymal stem cell derived microvesicles: Trophic shuttles for enhancement of sperm quality parameters. Reprod Toxicology. (2013) 42:78–84. doi: 10.1016/j.reprotox.2013.07.024, PMID: 23958892

[118] Huang J Li S Yang Y Li C Zuo Z Zheng R. GPX5-enriched exosomes improve sperm quality and fertilization ability. Int J Mol Sci. (2024) 25:10569. doi: 10.3390/ijms251910569, PMID: 39408895 PMC11477019

[119] Wang Y Liu Q Sun Q Zheng L Jin T Cao H. Exosomes from porcine serum as endogenous additive maintain function of boar sperm during liquid preservation at 17 °C *in vitro* . Theriogenology. (2024) 219:147–56. doi: 10.1016/j.theriogenology.2024.02.015, PMID: 38430799

[120] Shamsi RR Jozani RJ Asadpour R Rahbar M Taravat M. Seminal plasma-derived exosome preserves the quality parameters of the post-thaw semen of bulls with low freezeability. Biopreservation Biobanking 23:364–73. (2024). doi: 10.1089/bio.2024.0077, PMID: 39723439

[121] Zakrzewski W Dobrzynski M Szymonowicz M Rybak Z. Stem cells: past, present, and future. Stem Cell Res Ther. (2019) 10:68. doi: 10.1186/s13287-019-1165-5, PMID: 30808416 PMC6390367

[122] De Luca M Aiuti A Cossu G Parmar M Pellegrini G Robey PG. Advances in stem cell research and therapeutic development. Nat Cell Biol. (2019) 21:801–11. doi: 10.1038/s41556-019-0344-z, PMID: 31209293

[123] Yamaguchi N Horio E Sonoda J Yamagishi M Miyakawa S Murakami F. Immortalization of mesenchymal stem cells for application in regenerative medicine and their potential risks of tumorigenesis. Int J Mol Sci. (2024) 25:13562. doi: 10.3390/ijms252413562, PMID: 39769322 PMC11676347

[124] Frisbie L Buckanovich RJ Coffman L. Carcinoma-associated mesenchymal stem/stromal cells: architects of the pro-tumorigenic tumor microenvironment. Stem Cells. (2022) 40:705–15. doi: 10.1093/stmcls/sxac036, PMID: 35583414 PMC9406606

[125] Coffman LG Pearson AT Frisbie LG Freeman Z Christie E Bowtell DD. Ovarian carcinoma-associated mesenchymal stem cells arise from tissue-specific normal stroma. Stem Cells. (2019) 37:257–69. doi: 10.1002/stem.2932, PMID: 30353617 PMC6392140

[126] Rodríguez-Fuentes DE Fernández-Garza LE Samia-Meza JA Barrera-Barrera SA Caplan AI Barrera-Saldaña HA. Mesenchymal stem cells current clinical applications: A systematic review. Arch Med Res. (2021) 52:93–101. doi: 10.1016/j.arcmed.2020.08.006, PMID: 32977984

[127] Yuan FM Li YM Wang ZH. Preserving extracellular vesicles for biomedical applications: consideration of storage stability before and after isolation. Drug Delivery. (2021) 28:1501–9. doi: 10.1080/10717544.2021.1951896, PMID: 34259095 PMC8281093

[128] Qi J Zhou YL Jiao ZY Wang X Zhao Y Li YB. Exosomes derived from human bone marrow mesenchymal stem cells promote tumor growth through hedgehog signaling pathway” (vol 42 pg 2242, 2017). Cell Physiol Biochem. (2021) 55:805–6:pg 2242. doi: 10.1159/000479998, PMID: 28817816

[129] Vakhshiteh F Atyabi F Ostad SN. Mesenchymal stem cell exosomes: a two-edged sword in cancer therapy. Int J Nanomedicine. (2019) 14:2847–59. doi: 10.2147/IJN.S200036, PMID: 31114198 PMC6488158

[130] Qu Q Fu B Long Y Liu ZY Tian XH. Current strategies for promoting the large-scale production of exosomes. Curr Neuropharmacol. (2023) 21:1964–79. doi: 10.2174/1570159X21666230216095938, PMID: 36797614 PMC10514529

